# Genome-wide DNA methylation reprogramming in response to inorganic arsenic links inhibition of CTCF binding, DNMT expression and cellular transformation

**DOI:** 10.1038/srep41474

**Published:** 2017-02-02

**Authors:** Matthew Rea, Meredith Eckstein, Rebekah Eleazer, Caroline Smith, Yvonne N. Fondufe-Mittendorf

**Affiliations:** 1 Department of Molecular and Cellular Biochemistry, University of Kentucky, Lexington, KY 40536, USA; 2 Bellarmine University, Louisville, KY 40205, USA

## Abstract

Chronic low dose inorganic arsenic (iAs) exposure leads to changes in gene expression and epithelial-to-mesenchymal transformation. During this transformation, cells adopt a fibroblast-like phenotype accompanied by profound gene expression changes. While many mechanisms have been implicated in this transformation, studies that focus on the role of epigenetic alterations in this process are just emerging. DNA methylation controls gene expression in physiologic and pathologic states. Several studies show alterations in DNA methylation patterns in iAs-mediated pathogenesis, but these studies focused on single genes. We present a comprehensive genome-wide DNA methylation analysis using methyl-sequencing to measure changes between normal and iAs-transformed cells. Additionally, these differential methylation changes correlated positively with changes in gene expression and alternative splicing. Interestingly, most of these differentially methylated genes function in cell adhesion and communication pathways. To gain insight into how genomic DNA methylation patterns are regulated during iAs-mediated carcinogenesis, we show that iAs probably targets CTCF binding at the promoter of DNA methyltransferases, regulating their expression. These findings reveal how CTCF binding regulates DNA methyltransferase to reprogram the methylome in response to an environmental toxin.

Inorganic arsenic (iAs), is an environmental carcinogen with worldwide exposure to millions of people through water, and with health effects ranging from acute toxicities to malignant transformation^1^,^2^,^3^,^4^. The extent of adverse effects to humans relates to the dose and length of exposure^2^,^5^,^6^. Although these health risks are known, the molecular mechanisms that drive the toxicity and diseases mediated through iAs exposure are not well understood. Several possible mechanisms have been proposed including oxidative stress and genotoxic DNA damage^4^,^7^,^8^,^9^. However, recent evidence suggests that another even less understood, but equally important mechanism, is toxicity produced by changes in epigenetic-regulated gene expression.

In eukaryotes, genomic DNA is organized into nucleosomes, the fundamental repeating unit of chromatin^10^. Each nucleosome consists of 147 base pairs of DNA wrapping 1.7 turns around a histone octamer comprised of two copies each of H2A, H2B, H3, and H4^11^,^12^,^13^. The packaging of the genome with histones to form chromatin is essential for DNA condensation and protection. Likewise, changes in chromatin structure through epigenetic marks are essential for proper regulation of gene expression. Several studied have shown that iAs exposure alters epigenetic marks, such as histone post-translational modifications (PTMs), histone variants and DNA methylation, resulting in changes of gene expression patterns for both transcription initiation and gene splicing^4^,^14^. Of these epigenetic marks, DNA methylation is considered the most stable, and involves the addition or removal of methyl groups from the 5′ position of cytosine^15^,^16^. On the other hand, histone PTMs are more labile, maintained by a balance between histone modifying enzymes, which add or remove specific modifications.

In mammals, DNA methylation is located predominantly at CpG cites within the genome, with the majority of these sites being methylated. However this epigenetic mark also occurs at non-CpG sequences, CHG and CHH, where H = C, T or A^16^,^17^,^18^,^19^,^20^. Gene expression changes in response to several environmental toxins implicate alterations in DNA methylation^3^,^4^,^21^,^22^. Methyl groups are added to cytosine residues by the DNA methyltransferases (DNMTs). DNMT-3A and DNMT-3B regulate *de novo* methylation, early in development, while DNMT-1 is responsible for maintaining methylation status as cells replicate^23^,^24^. There is evidence that iAs alters DNA methyltransferase activity and subsequently DNA methylation patterns in cells^25^. Supporting this theory, are studies that show an overall DNA hypomethylation in cells that were exposed to iAs^4^,^25^,^26^,^27^,^28^. Studies also show decreases in expression of the methyltransferases, DMNT1 and DMNT3A, with low-dose iAs exposure^25^,^29^.

It is becoming increasingly apparent that aberrant methylation of gene promoter regions is the major mechanism of gene silencing in tumors. Aberrant DNA methylation, especially within CpG Islands (CGIs), appears to be a prevalent characteristic in many cancers, but other regulatory regions (CGI shores and enhancers) have been implicated in the differential expression of oncogenes and tumor suppressors^30^,^31^,^32^. Interestingly, though global hypomethylation has been observed in iAs-exposed cells, hypermethylation of specific genes has also been observed^33^,^34^. One study showed that 17 tumor suppressor genes, including p53, are hypermethylated in iAs-exposed cells, which leads to a reduced gene expression^28^,^33^. Such changes would have a great impact on the progression of the carcinogenic pathology. Although *in vitro* and *in vivo* studies have demonstrated that exposure to iAs modifies DNA methylation patterns, the genome-wide specific targets remain largely unknown^25^,^35^,^36^,^37^. We believe that studies of global changes in 5-methyl cytosine are not sufficient to understand the mechanisms that provoke carcinogenesis by iAs, and a more detailed gene specific analyses is important and warranted.

Others and we have shown that exposure to low dose of iAs results in epithelial-to-mesenchymal transition, a key step in cancer progression^14^,^38^. In this study, we investigated a potential epigenetic mechanism of chronic low dose arsenic exposure, DNA methylation, and examined target genes genome-wide in iAs-mediated EMT. We analyzed the change in DNA methylation levels during iAs-mediated EMT using high-resolution methylation sequencing (Methyl-Mini Seq), which maps methylation changes with single-nucleotide resolution. We observed changes in DNA methylation throughout the genome, and in all three cytosine methylation sequence contexts (CpG, CHG, and CHH), but focus this report on CpG methylation, as it is the predominant context for DNA methylation in mammals. We observed methylation and expression changes at key genes, suggesting a potential mechanism through which epigenetic methylation can drive differential gene expression of oncogenes and tumor suppressors. Such analyses are key to identifying possible target genes that propel the carcinogenic potential of iAs.

## Results

### iAs-induced genome-wide DNA methylomic alterations during iAs-transformation cells

We previously showed that iAs-mediated transformation resulted in chromatin structural changes^14^,^38^. We therefore hypothesized that iAs effects epigenetic changes resulting in alterations in gene expression. Indeed recent low-resolution studies reported iAs-mediated changes in methylation status and consequent altered expression of specific genes^39^. To extend these studies, BEAS-2B cells were transformed with iAs as previously described^14^,^38^ and global DNA methylation in normal cells (NT) and iAs-transformed cells (iAs-T) was assessed using the 5-methylcytosine (5-mC) DNA ELISA kit. Quantification of the 5-mC within the NT and iAs-T samples, showed a slight, though not significant, decrease in global DNA methylation in iAs-T cells compared to NT (Supplemental Fig. 1).

### Genome-wide analysis of DNA methylation changes

The measurement of global methylation using ELISA is a low-resolution technique, covering only a small percentage of the CpG sites, and therefore is unable to identify the genes targeting iAs-mediated differential methylation. Thus, to investigate the molecular effects of iAs on DNA methylomic signatures in iAs-T cells, we used the Methyl-MiniSeq EpiQuest system (an improved version of Reduced-Representation Bisulfite Sequencing (RRBS)), to measure, at a single-nucleotide resolution, the target genes of iAs-mediated methylation. Genomic DNA was isolated from NT and iAs-T cells, digested by restriction endonuclease enzymes TaqαI and MspI to produce CpG-rich fragments. These fragments were ligated to adapters, recovered, subjected to bisulfite conversion and sequenced. After sequencing, changes in methylation were quantified and used to determine the differential methylation patterns that may contribute to differential gene expression at the transcriptional and splicing levels in NT versus iAs-T cells. This dataset represents one of the most comprehensive genomic portraits of the impact of iAs on the DNA methylome, gene expression and iAs toxicity reported to date. From an analytical perspective, the dataset has considerable utility for developing pipelines to mine this sequence-based genomic data as well as to evaluate the relative contributions of methylation aberrations in defining patterns of gene expression in iAs-mediated transformation.

Methyl-MiniSeq was performed on two replicates of NT and iAs-T samples and results showed high concordance between replicates (Supplemental Fig. 2). From sequencing, the mapping efficiency to the GRCh37hg19 Reference Assembly was >5 0%. After filtering for non-unique reads we retained 8.74 × 10^6^ NT replica 1, 8.4 × 10^6^ NT replica 2, 8.67 × 10^6^ iAs-T replica 1. And 9.03 × 106 iAs-T replica 2 unique CpGs in NT and iAsT, respectively, which represents a greater than 6X average CpG coverage. Furthermore, there was greater than 98% conversion rate for both of samples, indicating that the false-negative rates were less than 2% (Supplemental Table 1).

### Profiling of DNA methylation in iAs-T cells

#### Chromosomal DNA methylation patterns

**Figure 1:**
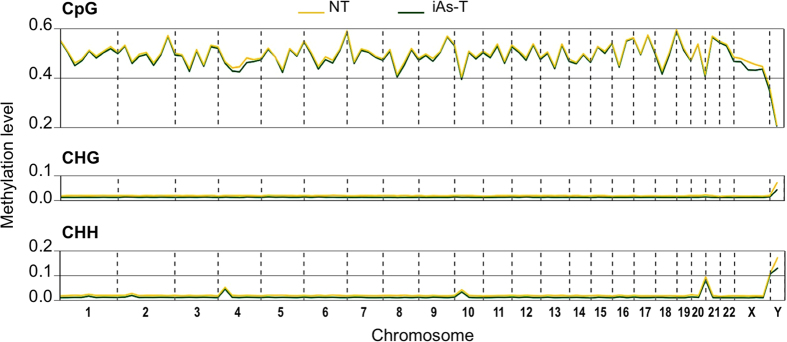
Chromosome views of methylation levels during iAs-induced transformation. Chromosome mapping of Methyl-MiniSeq CpG, CHG, and CHH shows there are no differences in DNA methylation at individual chromosomes.

To understand the DNA methylation events in iAs-T cells, we first profiled iAs-induced DNA methylation changes on individual chromosomes (Fig. 1). The extent of iAs-mediated DNA methylation on each chromosome was determined by calculating a fold-change in DNA methylation level in iAs-T cells vs NT cells. Our analyses showed that the methylation levels were similar between NT and iAs-T, even within replicates (Supplemental Fig. 2), across all chromosomes. These results indicated that the bisulfite conversion and Methyl-Mini Seq analyses were performed properly and/or that iAs-mediated methylation changes are not specific for any particular chromosome, but evenly distributed across the genome. Although we did not observe changes in the average level of DNA methylation per chromosome in iAs-T compared to NT samples, it is still possible that changes in DNA methylation occur at specific regulatory sites. Indeed, this is in line with previous studies showing lack of global DNA methylation changes, though with tissue/cell-type locus-specific DNA methylation changes^31^,^40^.

#### Average global DNA methylation at specific gene regulatory sites

**Figure 2:**
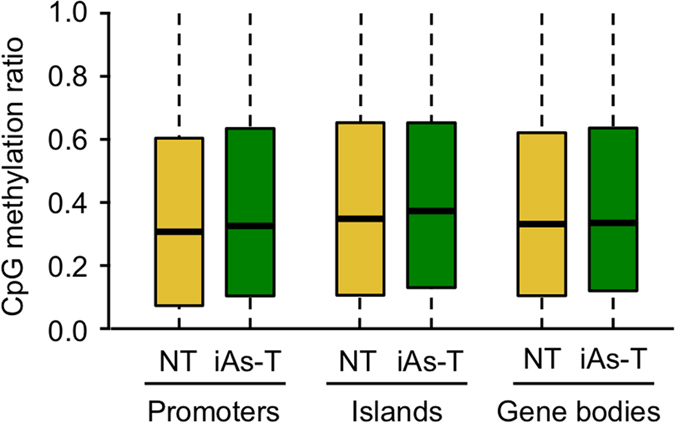
Analysis of CpG methylation levels at promoters, islands, and gene bodies. Global CpG methylation levels are not changed at promoter, islands and/or in gene bodies. The boxes denote the 25^th^ and 75^th^ percentile (bottom and top of box), and median value (horizontal band inside box). The whiskers indicate the values observed up to 1.5 times the interquartile range above and below the box.

We next measured the average DNA methylation at specific global genomic sites - promoters, CpG islands and gene body (introns and exons). Our analyses showed that there were no global changes for the average CpG methylation in any of these regions (Fig. 2, Supplemental Fig. 2b). We also analyzed the average global changes to CHG and CHH methylation contexts across the various gene regulatory regions. For both CHG and CHH contexts, we observed a slight but not significant increase in average global methylation at promoters, CGIs and gene bodies (Fisher’s test p-value < 0.05; Supplemental Figs 3 and 4).

**Figure 3:**
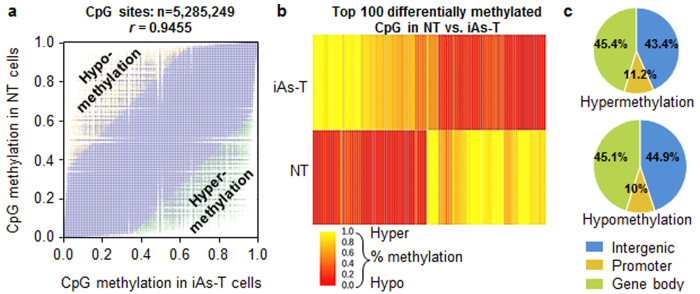
Differential CpG methylation patterns as cells undergo iAs-induced transformation. (**a**) Scatterplot of CpG methylation showing high correlation between samples, however differences are seen; (**b**) Methylation signal of differentially methylated CpG regions; (**c**) Distribution of CpG DMRs across top 2000 sites (intergenic, promoter, gene body [exon, intron]) in hypomethylated and hypermethylated contexts.

Though global CpG methylation (across chromosomes and average global content) showed no dramatic changes, we calculated and plotted the methylation changes at each CpG site (5.28 × 10^6^ CpG sites) between NT and iAs-T cells. Though we observed a high correlation between the two samples (Pearson correlation of *r* = 0.9455), there were however some outliers, showing hyper and hypo-methylation in iAs-T compared to NT samples (Fig. 3, Supplemental Fig. 2c). These data suggest that global measurements to changes in CpG methylation do not capture the complete picture. In fact, site-specific changes in DNA methylation drive specific gene expression patterns, which may not be recorded globally^41^. Additionally, our analysis of differential DNA methylation within the CHG and CHH contexts showed a lower correlation in methylation patterns between NT and iAs-T cells, compared to the correlation of CpG sites between the two samples. One possibility is that though there are many CpG sites in the genome and few of these sites have differential methylation changes. On the other hand, there are fewer CHGs and CHH sites genome-wide, and methylation changes at the sites will be more significant (Supplemental Figs 5 and 6).

#### Loci specific differential methylation

Our analyses showed that more than 12000 sites are strongly differentially DNA methylated in iAs-T cells (Fig. 3). We define strongly differentially methylated sites as those sites having changes greater than 33% (Fisher’s test, p value < 0.05, FDR q-value < 0.01) compared to the same sites in NT cells. From the outliers in Fig. 3a, we observed significant changes in CpG DNA methylation at 2000 loci (including intergenic, promoter, genic). Indeed the robustness of the changes in DNA methylation is clearly shown with a heatmap of the top 100 CpG differentially methylated regions (DMRs) (Fig. 3b, Supplemental Table 2). Next, to gain a functional understanding into these changes, we analyzed differential DNA methylation at genic as well as promoter regions. Promoter regions were defined as being +/− 1 kb from transcription start site, while genic regions (gene bodies) include exons and introns. Comparison of CpG hypermethylation patterns across different regulatory regions of individual genes revealed that only 11.2% of the differential methylation occurred at the promoters, while 45.4% occurred in genic regions and 43.4% occurred at intergenic regions. Likewise for hypomethylated regions, 10% were promoter regions, 45.1% genic and 44.9% intergenic regions (Fig. 3c).

Similar analyses were performed on the differential methylation of CHG and CHH sites (of which about 1000, were within genes) and the robust changes at the top 100 DMR sites were again plotted as heatmap (Supplemental Figs 5 and 6; Supplemental Tables 3 and 4). Likewise differentially hypermethylated CHG (Supplemental Fig. 5c) and CHH regions (Supplemental Fig. 6c) in iAs-T cells showed a pattern similar to that observed at CpG sites (Fig. 3), with more found in genic regions than intergenic regions and the least at promoters. This same pattern was observed for regions that became hypomethylated in iAs-T cells (Supplemental Figs 5c and 6c). This observed pattern is in line with previous studies of differential methylation at regulatory regions^42^,^43^,^44^.

#### Gene Ontology

To explore the biological functions associated with DMR-containing genes, we used the Gene Ontology program (PANTHER- GO SLIM) to identify enrichment of different Biological Processes (BP)^45^,^46^. For this analysis, genes within the top-2000 DMRs were used, as limiting our search to the topmost 100 would reduce the set of GO terms, and important gene families might not be detected. For CpG hypermethylated DMRs, 831 unique genes were identified within the top 2000 DMRs. These genes include GO terms of neural development, cell-cell signaling, cell communication and localization (Table 1). There were 865 unique genes within this top-2000 hypomethylated DMRs. Likewise, the hypomethylated genes showed over-representation in GO BP terms of cell adhesion, cell communication, nervous system and cell adhesion (Table 1). In summary, since hyper- and hypo-methylated DMRs, targeted genes involved in the same GO BP terms, the possibility that these pathways are targeted during iAs toxicity and transformation seems reasonable. For the genes with differentially methylated CHG and CHH sites, the GO terms (BP) that were most over-represented were genes involved in, cell adhesion, cell communication, and nervous system development (Supplemental Tables 5 and 6). In summary, a large number of genes with both hyper and hypo-DMRs in iAs-T cells, were found to be associated with cell communication and cell adhesion, which could indicate that iAs targets these pathways to drive its carcinogenic potential.

To further understand the epigenetic mechanism of iAs mediated methylation changes, we carried out additional analyses using GSEA to determine if there is any correlation between H3K37me3 and the identified genes with DMRs^47^. The reasoning behind this analysis is that there is interplay between DNA methylation and H3K27me3^48^,^49^. For this analysis we used only the genes with differential methylation at their promoters since H3K27me3 is promoter-specific. This analysis showed an over-representation of iAs-target genes as also being targets of H3K27 trimethylation and components of the Polycomb repressive complex, PRC2 (Table 1)^47^,^50^.

#### Validation of Methyl-seq DNA methylation changes

**Figure 4:**
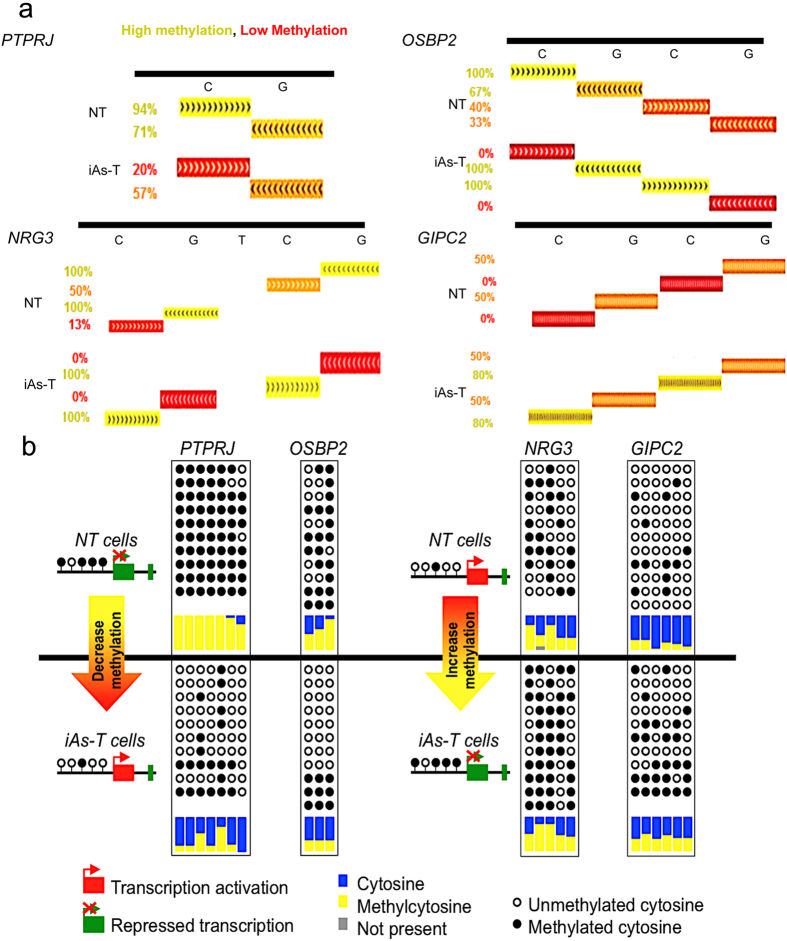
Validation of methyl-seq. Bisulfite conversion and pyrosequencing of specific gene promoter loci to confirm Methyl-MiniSeq data. (**a**) DNA methylation data from Methyl-MiniSeq study are shown using a UCSC browser track displaying our data set. High methylation in yellow and low methylation in red. (**b**) Graphical representation of methylation at each promoter or gene body sites. Lollipop representation of bisulfite converted DNA (NT on top iAs-T on bottom). PTPRJ (Chr11:48001742), OSBP2 (Chr22:31091206), NRG3 (Chr10:83634392), GIPC2 (Chr1:78511849). Schematic shows DNA hypo or hyper methylation in promotor region of representative genes and the impact of the methylation status on transcriptional activity. Arrow indicates the direction of differential methylation changes from normal (NT) cells to iAs-transformed (iAs-T) cells.

To validate the changes in DNA methylation seen from the Methyl-Mini Seq (Fig. 4a), we chose two genes with hypermethylation at the promoter region and two genes with hypomethylation within the promoter region. Genomic DNA from NT and iAs-T cells were Bisulfite converted using the EZ DNA Methylation-Lightning Kit. About 300 bp fragments surrounding the target regions were PCR amplified, cloned and sequenced. One example is the DMR in the promoter of *PTPRJ*, which showed promoter hypomethylation at the CpG site - Chr11:48001742, in our Methyl-Mini Seq analyses. After bisulfite conversion, cloning and pyrosequencing, we observed that this locus was hypomethylated (Fig. 4b). In addition to this CpG site, hypomethylation was observed at other adjacent CpG sites in iAs-T cells. A second example showing hypomethylation after iAsT is in the promoter of *OSBP2* (Chr22:31091206) (Fig. 4b). We next tested two other genes that showed high hypomethylation in NT cells that became hypermethylated in iAsT cells. The first example is at the specific CpG site (Chr10:83634392) of the gene *NGR3*. Using, pyrosequencing, we confirmed that this region is indeed hypermethylated in iAs-T cells. As with the *PTPRJ* gene promoter region, other CpG sites in the vicinity were also hypermethylated (Fig. 4b). The second example with an increase in methylation after iAs-mediated transformation is at the promoter of the *GIPC2* gene. Here too, pyrosequencing confirmed the hypomethylation pattern in NT cells, and also that this methylation pattern became altered in iAs-T cells. Although Methyl-Mini Seq identified only one hypermethylated site at the promoter of *GIPC2* -Chr1:78511849, pyrosequencing showed that hypermethylation also occurs at nearby CpG sites within the region (Fig. 4b). These results indicate that our genome-wide methylation studies are reproducible and were validated by pyrosequencing.

#### Differential methylation is coupled to differential gene expression at the transcription initiation and splicing levels in iAs-induced transformation

**Figure 5:**
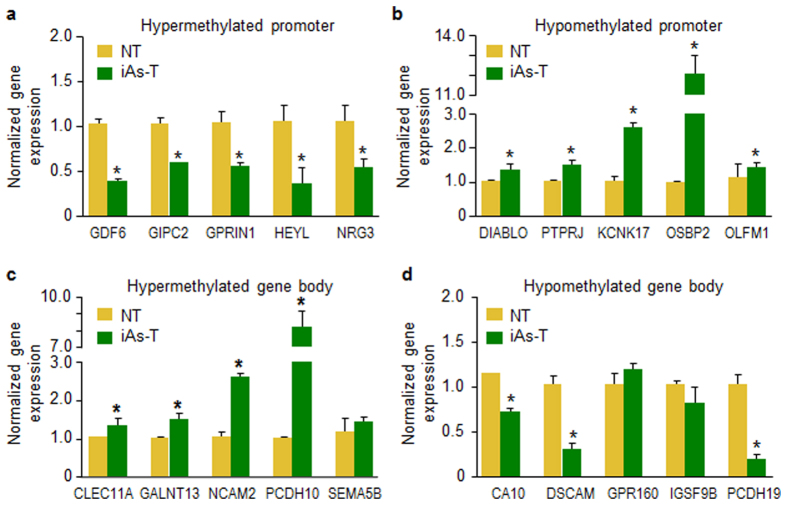
Correlation of differential methylation and gene expression. Gene expression changes in top CpG DMR associated with genes. qRT-PCR for changes in gene expression at top CpG DMRs: (**a**) Hypermethylated promoters, (**b**) hypomethylated promoters, (**c**) hypermethylated gene body, (**d**) hypomethylated gene body. All were performed in triplicate and normalized to GAPDH expression. Error bars are S.E.M.

Promoter DNA methylation status has been linked to repression of gene expression. While the function of DNA methylation in gene bodies is not as clear as for promoter methylation, it has been linked to the activation of gene expression. To examine the influence of DNA methylation on gene expression, we analyzed the expression of some genes differentially methylated at the promoters (Supplemental Tables 7 and 8). We performed qRT-PCR to investigate the expression level of these genes. For these analyses, we chose several genes that had differential promoter methylation using Methyl-Mini Seq and confirmed by Bisulfite Conversion and pyrosequencing (Fig. 4). For example, we showed that the promoter region of *NRG3* was differentially methylated and measurement of the gene expression at this promoter showed a reduction in gene expression (Fig. 5a). In contrast genes that are promoter hypomethylated after iAs-T showed an increase in expression levels, including *PTPRJ* (Fig. 5b). Since several genes showed gene body DNA differential methylation, we also analyzed their gene expression. For genes with hypermethylation in the gene body - *CLEC11A* and *PCDH10*, we observed an increase in gene expression (Fig. 5c). On the other hand, genes with gene body hypomethylation - *DSCAM* and *PCDH19*, showed a general decrease in gene expression (Fig. 5d). Next, to determine to what degree the DNA methylation changes effect gene expression differences in NT and iAs-T cells, we performed gene expression microarray analyses on NT and iAs-T cells. From the significantly differentially methylated genes, about 36% of the genes (706) showed differences in DNA methylation also showed a change in gene expression (p < 0.05). Interestingly, the Gene ontology of these genes within these category, showed similar GO terms to genes with DMRs only (such as Neurogenesis, cell adhesion, etc.- Supplemental Table 9).

**Figure 6:**
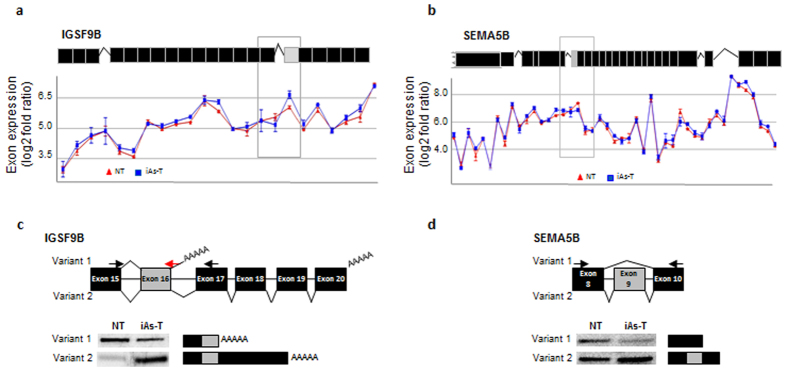
Changes in alternative splicing patterns in iAs-mediated transformation. Cartoon images of the genes (above) and analysis of exon expression levels (log_2_) from array probes against *IGSF9B* (**a**) and *SEMA5B* (**b**). Validation of differential splicing changes in *IGSF9B* (**c**) and *SEMA5B* (**d**) above are zoomed cartoon images of possible variants exons. Gray boxes indicate areas where alternative splicing changes are seen.

We observed that not all gene body methylation changes, resulted in a change in gene expression. For example *SEMA5B*, which becomes hypermethylated and *IGSF9B* which becomes hypomethylated with iAs-mediated transformation showed no change in gene expression when levels were compared between NT and iAs-T cells (Fig. 5c and d). This finding is not surprising as our understanding of function of gene body methylation is still limited. Gene body methylation can function in other ways such as in the regulation of alternative splicing rather than the regulation of gene transcription initiation^51^,^52^,^53^. To test for this hypothesis, we again used the Affymetrix human transcriptome 2.0 ST microarray analyses, which is able to detect both gene expression and splicing changes. Analyses from this array indeed showed differential splicing patterns in these two genes (Fig. 6a,b). We further used semi-quantitative PCR to validate these splicing differences. For instance, at IGSF9B, we observed that there was a decrease in ‘variant 1’ expression and an increase in ‘variant 2’ in iAs-T (Fig. 6c). In SEMA5B gene, there is exon inclusion (exon 9) while in NT, this exon is excluded (Fig. 6d). We next sought to understand the correlation between DNA methylation and alternative splicing within these two samples (NT vs. iAs-T). As a whole, we observed 772 genes that were alternatively spliced and had changes in DNA methylation (promoter or gene body), of which 454 had changes in gene expression as well (Supplemental Fig. 7). Thus narrowing to only changes in alternative splicing showed that about 16% of the genes (318) that had changes in DNA methylation levels showed a change in alternative splicing events (p < 0.05). Similar to the gene expression data, these overlapping genes had similar GO terms (Supplemental Table 9) such as cell projections, neural development and cellular localization, indicating the possibility that these pathways are targeted by iAs for cellular transformation. Overall, we believe that the overlap between gene expression/splicing and DNA methylation in our data sets, is a likely a conservative estimate because of the technical differences in protocols and the inherent loss of information in threshold and significance threshold calls.

### Reduction of CTCF occupancy at DNA methyltransferase promoters occurs during iAs-mediated EMT

**Figure 7:**
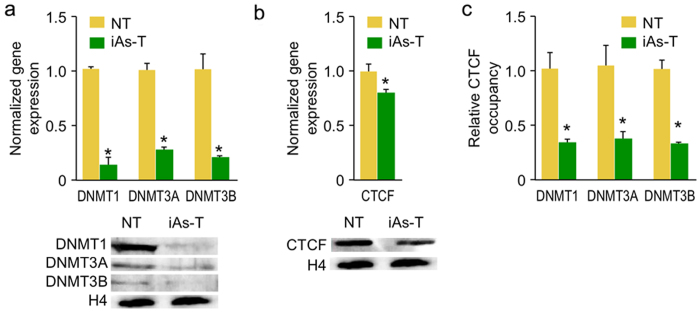
Depletion of DNMTs correlates with reduced CTCF binding during iAs-meditated transformation. (**a**) qPCR of DNMT cDNA (upper) shows a depletion in gene expression, which is confirmed by western blot analysis (lower). (**b**) qPCR (upper) and western blot analysis (lower) showing that the transcription factor CTCF is downregulated (about 20% in iAsT) during iAs-induced transformation. (**c**) Chromatin immunoprecipitation shows that CTCF binds significantly less at the three DNMT promoter regions during iAs-mediated EMT. All qPCR were performed in triplicate and gene expression was normalized to GAPDH expression, while ChIP occupancy studies were normalized to the 1% of Input. Error bars are S.E.M.

It is hypothesized that reduced DNA methylation occurs through two non-mutually exclusive mechanisms: (1) the competition of methyl groups by arsenic methyl-transferase-3 needed for the metabolism of arsenic and (2) reduction of the expression DNA methyltransferases. Indeed, in low-resolution studies a dose-dependent decrease in the DNMT1 and DNMT3A expression has been reported in response to iAs^25^. We therefore investigated whether DNMTs were affected at both the transcription and protein levels in response to iAs-transformation. We first measured and compared the transcript levels of three important DNMTs (*DNMT1, DNMT3A and DNMT3B*) using quantitative real-time PCR (qRT-PCR). From these analyses, we observed a significant decrease in the transcript levels of these DNMTs in iAs-T compared to NT cells (Fig. 7a, upper). Next, we asked whether these changes also occur at the protein level. For this analysis, we measured the protein levels of these DNMTs by western blot and a decrease in protein levels for DNMT1, DNMT3A and DNMT3B were observed (Fig. 7a, lower).

Next we examined how iAs mediates the expression of DNMTs. Analysis of the promoter region of the DNMTs, using the UCSC browser (mod ENCODE data) showed binding sites for CCCTC-binding factor (CTCF). CTCF is a highly conserved zinc finger protein, best known as a transcription factor. However, the protein is also known to play a role in DNA methylation; it can function as a transcriptional activator, a repressor or an insulator, blocking the communication between enhancers and promoters. Interestingly, iAs has been shown to inhibit CTCF binding to DNA^28^,^54^. We therefore considered if the CTCF could act as a TF at DNMT promoters^55^, and if so, in its absence DNMTs would not be expressed. To answer this question, we first determined if a reduction in CTCF expression was observed in iAs-T cells. This information was critical as our Methyl-MiniSeq sequencing showed hypomethylation within the gene body of CTCF (Fig. 7b). Using qRTPCR and western blot analyses, we observed a reduction of about 20% in iAs-T cells (Fig. 7b). It is possible that the reduction in CTCF expression results in less CTCF occupying the DNMT promoter. We therefore measured the relative occupancy of CTCF at the DNMT promoters using chromatin immunoprecipitation with anti-CTCF antibody followed by quantitative real time PCR (ChIP-qPCR). These analyses showed that at all three DNMT promoters, the relative CTCF occupancy (normalized to IgG control) showed a 64–67% reduction in the iAsT cells compared to NT cells (Fig. 7c). As negative control for a non-CTCF binding site, we used measured occupancy of CTCF within DNMT1 gene body. Relative occupancy of CTCF within this site (gene body) was not changed between NT and iAs-T samples and was near background levels (data not shown). If we assume that a 20% reduction of CTCF occupancy is caused by reduction in CTCF expression, there is still a 40% reduction in occupancy that cannot be explained by expression levels alone. We interpret this 40% reduction of CTCF occupancy as due to an inhibition by iAs to its DNA binding. In iAsT cells, a reduction in CTCF occupancy at these promoters in iAs-T cells, might therefore play a role in the repression of DNMTs.

## Discussion

This study took a genome-wide approach to investigating changes in DNA methylation as cells undergo iAs-mediated EMT. We present genome-wide DNA methylation profiles of iAs-induced EMT in BEAS-2B cells, allowing a characterization of the extensive reprogramming of the methylome that occurs during this transition. While many other studies have carried out similar studies by analyzing this important epigenetic mark in response to iAs exposure, most have analyzed DNA methylation as a global event or only in specific gene-specific contexts. This study uses the power of Methyl-seq to find changes in DNA methylation at base pair resolution, which provides (1) an unbiased coverage of the entire genome at high resolution and (2) quantification of methylation changes at nucleotide resolution. Our analysis shows that DNA methylation changes appear to play an important role in the gene expression changes that occur during the EMT process in the lung epithelial cell line BEAS-2B. The changes seen were not at a global or even chromosome scale (Fig. 1, Supplemental Fig. 2a), but were only seen once individual loci were analyzed with the Methyl-sequencing of millions of CpG sites (Figs 2 and 3). Through Methyl-MiniSeq we are able to analyze regions with differential methylation changes at high resolution, and our results show that DNA methylation is a dynamic process during iAs-induced carcinogenesis; hypermethylation at specific regions, while also modulating hypomethylation at other specific sites. The genes targeted by iAs during this EMT transition are involved in cell-communication and changes in their gene expression, may drive the carcinogenic potential of iAs^25^ (Table 1, Supplemental Tables 5,6,9). The mechanisms by which these important epigenetic regulators are changed with iAs exposure are unclear.

### What genes are targeted in differential methylation during iAs-induced carcinogenesis?

From our results, we observed that both hyper- and hypomethylation at target genes (Fig. 4) with correlating consequences in gene expression (Fig. 5) and alternative splicing (Fig. 6). Many of the iAs-mediated DM gene targets are involved in cell junctions, cell adhesion, cell-cell communication and focal adhesion. Alterations in the expression of specific cell adhesion molecules are a common event in cancer, as loss of cell-cell and/or cell-extracellular matrix adhesion promotes cell growth as well as tumor dissemination. Thus cell adhesion molecules can be considered tumor suppressors since some are involved in limiting tumor cell migration^56^,^57^. The effect of cell adhesion in carcinogenesis is not as clear-cut as it seems because some of these genes are upregulated during carcinogenesis while others are downregulated (Table 1, Supplemental Tables 5,6,9). However, the change of gene expression for some of these adhesion genes has been implicated in carcinogenesis. Indeed, during iAs-mediated transformation, we observed a loss of epithelial markers (Claudins, Slug) and an increase in mesenchymal markers (Vimentin and N-cadherin)^14^,^38^. Therefore, it is possible that the epigenetic modulation of gene expression patterns involved in adhesion/EMT is a mechanism for iAs-induced carcinogenesis. Indeed, E-cadherin, which is downregulated in mesenchymal phenotypes, is silenced through both increased DNA methylation and H3K27me3 of the PRC2 complex^14^,^38^,^56^,^58^,^59^. Genes in both the Notch (*HEYL, NOTCH2/3 and CUT*) and Wnt (*CTNN*) pathways have been implicated in the regulation of the EMT process and a subset of genes from our analysis were differentially methylated. These changes to important pathways could push cells toward the mesenchymal phenotype, which then leads to the observed pathogenic changes.

### How does iAs reprogram DNA methylation patterns during pathogenesis?

One possible mechanism is that iAs modulates the expression of the DNA methyltransferase^60^,^61^,^62^. Indeed our results, supports this possibility as we observed a decrease in transcript and protein levels of all three methyltransferases (Fig. 7a and b) involved in DNA methylation (de novo as well as maintenance methylation marks). Our results are in line with previous studies reporting decreases in DNA methyltransferases in response to iAs exposure^60^,^61^,^62^. This mechanism is also supported by our results probing the transcription factor CTCF (Fig. 7c). Our results demonstrate a slight decrease in expression of CTCF with a large reduction in the relative occupancy of CTCF at the promoters of DNMTs in iAs-T cells. The large decrease in occupancy could also result from an inhibition of CTCF binding to its DNA target sites^54^; this inhibition in CTCF binding at the promoters of DNMTs results in a reduction of gene expression of DNMTs, with consequences for overall DNA methylation patterns. Inhibition of CTCF DNA binding, is in line with studies that show that iAs binds zinc finger-binding proteins with 3 Cysteine and 1 Histidine (C3H1) or C4 configurations and inhibit their DNA binding^63^,^64^. CTCF on the other hand, has 11 zinc-fingers that can bind to DNA in different combinations to effect its function^65^. While the first 10 zinc fingers are of the C2H2 type, the 11th finger is a C3H1 type^65^ (PDB 1X6 H) (Supplemental Fig. 8), suggesting a possible mechanism of DNA binding inhibition of CTCF by iAs. Thus, the modulation of CTCF and possibly other TFs within specific regulatory complexes could determine the specificity of gene regulation. For example, CTCF is known to recruit a variety of different complexes, and depending on the particular complex, CTCF can act as a TF, an activator, a repressor or an insulator (Reviewed by Kim)^66^. Furthermore, CTCF is also known to activate poly (ADP) ribose polymerase (PARP1), to prevent DNA methylation at specific sites^67^. Since CTCF could act as a TF or a DNA methylation modulator our findings of reduced CTCF occupancy at the DNMT promoters would be in line as its function as a TF in regulating gene expression. Especially since our genome-wide analysis did not show any methylation changes at the DNMT promoters.

Epigenome changes result in the structural rearrangement of chromatin with the consequence of alteration in transcription factor accessibility to target genes. Another possibility by which iAs is controlling the expression of the DNMTs could be this inhibition of TF binding to specific sites. iAs inhibits the binding of zinc finger proteins, some of which are transcription factors and important in regulating gene expression. Through inhibiting the TF from binding to DNA or modulating the epigenome (DNA methylation) to control the accessibility of the TF to their cognate sites. We asked whether some of these genes with DMRs were controlled generally by specific transcription factors^63^,^68^. Further Gene Ontology analysis showed that a number of the genes with differential methylation are potentially regulated by a small group of transcription factors (TFs). These TF include TCF3, NFAT, NFATC, MAZ, MLLT7, LEF1, REPIN1, and MYOD1, which control at least 70 genes across all of the DNA methylation pattern contexts - CpG, CHG, CHH, in both hyper and hypomethylation contexts. Many of these TF have been implicated in iAs response and in carcinogenesis^38^.

### Does iAs exposure affect other epigenetic marks?

Several studies report significant crosstalk between DNA methylation and histone PTMs. Specifically, there is considerable evidence for crosstalk between several histone PTMs and DNA methylation, especially H3K27me3 and DNA methylation^48^,^49^. Furthermore, studies in environmental epigenetics have also shown this interplay between DNA methylation and H3K27me3 in response to iAs. For instance, it was reported that during iAs exposure, both DNA methylation and H3K27me3 are co-regulated^48^,^69^,^70^. Interestingly in our analyses, we observed that many of the differentially methylated iAs target genes are also known H3K27me3 targets (Table 1). Though the effect of iAs on H3K27me3 was not studied directly, it is possible that the deposition of specific histone modifications and DNA methylation are being dynamically regulated during iAs-mediated EMT. Future studies are needed to identify any co-regulation of DNA methylation and specific histone PTMs during iAs-mediated EMT, specifically, H3K27me3. In depth analysis of the gene sets also revealed that a large number of these genes are controlled by a small set of transcription factors. Interestingly, some of these transcription factors themselves show differential methylation, which could impact many target genes as their expression could be changed with the methylation levels.

In summary, our results present the first comprehensive analyses of differentially methylated targets in iAs-mediated transformed cells. We also provide correlative analysis of differential methylation changes with the functional consequences in gene expression. This dataset represents one of the most comprehensive genomic portraits of the impact of iAs on DNA methylation changes and linked changes in gene expression reported, to date. Indeed, cell communication genes are the targets for differentially methylated genes during iAs transformation suggesting that these alterations could drive the carcinogenic potential of iAs. Finally, since many cell communication genes are targeted in iAs-differential methylation pathway, our results set a platform for the diagnosis and potential development of therapeutics required for iAs-mediated pathology.

## Methods

### Bisulfite conversion and pyrosequencing

To analyze the DNA methylation of the candidate genes, freshly isolated genomic DNA was digested with HindIII. 1 μg of digested DNA was used in bisulfite conversion according to the instructions with the kit (EZ DNA Methylation-Lightning Kit –Zymo Research). Converted DNA was eluted in 12 μl, and 2 μl was used in PCR reactions. Primers were designed using Zymo Research Bisulfite Primer Seeker (http://www.zymoresearch.com/tools/bisulfite-primer-seeker) around the CpG sites identified by Methyl-Mini Seq as being differentially methylated (Supplemental Table 10). PCR conditions for Bisulfite using ZymoTaq PreMix were as follows: (1) 95 °C 10 min; (2) 95 °C 30 sec; (3) 50–58 °C (dependent on primer pair) 40 sec; (4) 72 °C 45 sec; (5) repeat steps 2–4 for 40 cycles; (6) 72 °C 7 min. After amplification, PCR products were separated on a 1.5% agarose gel, and appropriate bands were cut and purified. Purified PCR product was then blunt-end modified (End-It DNA End-Repair Kit- Epicentre), and subcloned into pUC19 vector predigested with HincII. 10–15 clones from each product were sequenced (University of Chicago Comprehensive Cancer Center DNA Sequencing and Genotyping Facility). Sequences were aligned with the genomic sequence using BiQ Analyzer. This program aligns the sequences, determines the converted Cytosine residues and calculates percent methylation at specific CpG sites in the sequence.

### Methyl-MiniSeq library construction

Methyl-MiniSeq is an improved version of reduced representation bisulfite sequencing (RRBS), a service provided by Zymo Research. Libraries were prepared from 200–500 ng of genomic DNA digested with 60 units of TaqαI and 30 units of MspI (NEB) sequentially and then extracted with Zymo Research (ZR) DNA Clean & Concentrato-5 kit (Cat#: D4003). Fragments were ligated to pre-annealed adapters containing 5′-methyl-cytosine instead of cytosine according to Illumina’s specified guidelines. Adaptor-ligated fragments of 150–250 bp and 250–350 bp in size were recovered from a 2.5% NuSieve 1:1 agarose gel (Zymoclean Gel DNA Recovery Kit, ZR Cat#: D4001). The fragments were then bisulfite-treated using the EZ DNA Methylation-Lightning Kit (ZR, Cat#: D5020). Preparative-scale PCR was performed and the resulting products were purified (DNA Clean & Concentrator - ZR, Cat#D4005) for sequencing on an Illumina HiSeq.

### Alignment of sequencing reads from bisulfite-treated DNA

Raw sequencing reads were processed to trim sequencing adapters, low quality bases (Illumina 1.5, Q < 67) at the 3′ end and ambiguous bases in both ends using in-house scripts. A reference genome database was constructed using the human GRCh37/hg19 reference genome by predicting 40–350 bp sized Msp1-Taqα1 fragments, *in silico*. This reference data base was used to map the trimmed sequence reads obtained from Methyl-MiniSeq. This reference genome then had all C’s and G’s converted to T’s and A’s, respectively, indexed, which allowed for 2 different reference databases to compare sequence reads. Bowtie software was used to map and align the Methyl-MiniSeq sequencing reads to the both of the reference genomes^71^. Mismatches between the unconverted read and reference genomes in the alignment were counted, and loci in which a T in the Methyl-MiniSeq read was matched to a C in the unconverted reference were ignored. A read was considered unique only if the second-best alignment had 2 more mismatches, if other alignments of a read had less than 2 mismatches it was considered non-unique and discarded. To determine the methylation levels in each read the number of reads with a C for a locus was divided by the total number of reads with a C or T for that locus. The methylation of each site was determined as the fraction of methylated reads and unmethylated reads combined. Methylation sites with <5 reads were removed from further analysis and Fisher’s exact (t−) test was used for significance of methylation levels between NT and iAs-T samples. These analyses were performed in software pipelines using Python. Pearson’s product-moment coefficient, using script in R, was used to calculate correlation between the methylation across the two samples.

### Detection of DMRs

To further analyze the Methyl-MiniSeq sequencing data, the genome was divided into non-overlapping windows of 200 bp to characterize differentially methylated regions. Profiling windows that had less than 3 sites were discarded and those with more than 3 were used in further analysis. In each of the windows that were retained, the number of methylated or unmethylated site observations was calculated by adding the total number of sites in all reads to each window, and a Fisher’s exact test was used to assign p-values for each of these sites. When p-values were calculated for each of the sites, multiple-testing correction was performed for each 200-bp window separately, an FDR q-value was used to determine significance of the DMR^72^. To find the regions that were differentially methylated, when comparing NT to iAs-T, the methylation status of the 200-bp windows were determined and compared. For regions to be defined as differentially methylated, three criteria were needed to be met: (1) statistical significance (from Fisher’s test; p < 0.05), (2) FDR q-value < 0.01, (3) methylation levels had to change at least 25%. To obtain the DMRs between two groups, i.e., NT samples vs. iAs-T cells, we first identified the DMRs between the NT sample and the iAs-T sample by applying Fisher’s exact tests using the same stringent cutoff values in a pair wise fashion. Next, we selected only the common DMRs that were consistently identified in each NT sample compared with the iAs-T sample. Finally, the DMRs discovered in each NT sample when compared with the iAs-T sample were merged to make up the DMRs between two groups.

### Statistical analysis of Methyl-MiniSeq

Fisher’s t-test analysis was used to identify differentially methylated genes with statistical significance between groups (i.e., NT vs. iAs-T sample). The average methylation value of each window was used to perform the statistical analysis in R. The p-value was adjusted using FDR based on the method described above^38^. An FDR q value < 0.05 and a methylation difference >0.25 were the cut off values used to identify the statistically significant differentially methylated genes.

### RNA extraction and Array hybridization

Total RNA was isolated from cells using the RNeasy Kit (Qiagen) and quality assessment was conducted using RNA 6000 Nano-LabChip (Bioanalyzer, Agilent). Labeling of RNA and hybridization to the chip were performed at the University of Kentucky microarray core facility. RNA samples were labeled and hybridized to the Affymetrix Human Transcriptome 2.0ST array (HTA2), using one array per RNA sample. This array is able to highlight spliced isoforms of RNA using probes for both exons and exon-exon junctons, that are able to measure regions that may be excluded or included. The Affymetrix 3000 7 G scanner was used to quantify the signal intensity of hybridized probes, and data was processed using Command Console software version 4.1.2.

#### Gene level analyses

Initial gene expression patterns were identified as follows: intensities of the scanned arrays (CEL) of the 4 samples were imported using the GCRMA algorithm into Partek Genomics Suite 6.6 (Partek, MO). Genes were assembled for statistical analysis of expression at the gene-level by the array exon probes to calculate significant differential expression patterns using 1-ANOVA, followed by paired comparisons between the samples. A more detailed analysis of the data was performed as follows: Gene level analysis required probe set summarization and normalization. Raw ‘CEL’ files were processed using the Affymetrix Expression Console Software (build 1.3.1.187), normalized with “Gene Level – Default: RMA-Sketch” to create ‘CHP’ files for each of the samples. The CHP files are imported into the Affymetrix Transcriptome Analysis Console (TAC) 2.0 (build 2.0.0.9), using the option ‘Gene Level Differential Analysis’. Two conditions were created within the TAC (NT and iAs-T). The two replicates for each condition were loaded and the ‘Run Analysis’ step was performed to identify differentially expressed transcript clusters. Transcript clusters annotated using Affymetrix, and similar probe sets, do not have a one-to-one correspondence with protein coding genes, but may have multiple probes for each cluster or gene. Transcript clusters that include non-coding RNAs (lincRNA, snoRNA, miRNAs) have correspondence to one-to-one relationship between probes and genes. ANOVA (p < 0.05) and log2 fold change of ±1.2 (determined by Turkey’s bi-weight average) was used to compare each of the differentially expressed transcript clusters to NT. Large numbers of false positive differentially expressed genes could be found if only the use of p-value cutoffs are used in analysis, potentially underestimation of the variance. To account for this, a fold change (FC) cutoff was also incorporated to reduce the FDR. The choice of a log2 FC cutoff off of 1.2 (FC ± 2.3) reduced the set to a manageable size, allowing for meaningful interpretation.

### RNA transcript alternative splicing analysis

Splicing variants of the RNA transcripts were analyzed using the Partek Genomics Suite 6.6 (Partek, MO). HTA2 array data files (CEL) were imported and normalized using the GCRMA algorithm. Exon probes were summarized into genes and an ANOVA-1-way was used to determine alterative splicing between the NT and iAs-T samples. Statistical significance for splicing was p < 0.05.

### Alternative Splicing Analysis through semi-quantitative PCR

cDNA was prepared as above. 25 ng of the cDNA was used in the 25 μl PCR reactions. The reaction protocol was as follows: (1) 94 °C 5 min; (2) 94 °C 30 sec; (3) 54 °C (IGSF9B) or 57 °C (SEMA5B- See Supplemental Table 10) 30 sec; (4) 72 °C 45 sec; (5) repeat steps 2–4 for 29 total cycles; (6) 72 °C 10 min. 5.5 μl of 6X loading dye was added and 20 μl of each reaction was run on a 2% Agarose gel at 120 V for 2 hours. Gels were imaged on GE-Typhoon FLA9500.

## Additional Information

**Accession codes:** The datasets generated during and/or analyzed during the current study are available in the NIH GEO repository with accession number GSE85012.

**How to cite this article**: Rea, M. *et al*. Genome-wide DNA methylation reprogramming in response to inorganic arsenic links inhibition of CTCF binding, DNMT expression and cellular transformation. *Sci. Rep.* **7**, 41474; doi: 10.1038/srep41474 (2017).

**Publisher's note:** Springer Nature remains neutral with regard to jurisdictional claims in published maps and institutional affiliations.

## Supplementary Material

Supplementary Files

## Figures and Tables

**Table 1:** Gene Ontology of top DMRs associated with Genes.

GO Term	Number of Genes	p-value
**Hypermethylation** (**831 Genes**)		
Cell Communication	266	1.96E-04
Signal Transduction	235	3.63E-02
Metal ion binding	207	3.22E-02
Signaling	180	1.45E-05
Nervous system development	164	1.39E-13
Localization	145	1.05E-03
H3K27me3	106	4.69E-48
Neuron Generation	104	4.50E-07
SUZ12 Targets	100	6.30E-46
Cell-cell signaling	80	2.35E-04
H3K4me3 and H3K27me3	73	7.26E-41
Sequence-specific DNA binding	69	4.35E-02
PRC2 Targets	63	3.77E-29
Neurogenesis	60	9.44E-05
Neuron Differentiation	53	1.16E-04
Neuron Projection Development	49	1.44E-03
**Hypomethylation** (**856 Genes**)		
Localization	521	3.83E-02
Cell Communication	278	9.12E-05
Signaling	277	2.67E-05
Nervous system development	156	3.01E-09
Neurogenesis	111	1.15E-06
H3K27me3	105	7.04E-46
Cell Movement	89	7.00E-03
cell adhesion	88	5.41E-03
H3K4me3 and H3K27me3	86	1.50E-32
SUZ12 Targets	75	1.54E-25
Neuron Differentiation	74	1.11E-04
Cell-cell adhesion	64	3.04E-02
Neuron Development	56	2.95E-02
PRC2 Targets	52	7.36E-20
Neural Projection	42	6.73E-04
Transcription Factor activity	32	2.17E-02
Axon Guidance	27	1.45E-03
