# Supplementary material for: Genome-wide DNA methylation reprogramming in response to inorganic arsenic links inhibition of CTCF binding, DNMT expression and cellular transformation
Source: Sci Rep. 2017 Feb 2;7:41474. doi: 10.1038/srep41474 (PMC5288714; doi:10.1038/srep41474)
Supplement: Supplementary Files [file srep41474-s1.pdf]

Genome-wide DNA methylation reprogramming in response to inorganic arsenic links inhibition of CTCF binding, DNMT expression and cellular transformation

Matthew Rea[1], Meredith Eckstein[1], Rebekah Eleazer[1], Caroline Smith[1, 2], Yvonne N. Fondufe-Mittendorf[1]*

1. Department of Molecular and Cellular Biochemistry, University of Kentucky, Lexington, KY 40536, USA

2. Bellarmine University, Louisville, KY 40205, USA

***Corresponding Author:** y.fondufe-mittendorf@uky.edu

SUPPLEMENTAL INFORMATION

Supplemental Methods

Cell Growth and transformation with sodium arsenite. BEAS-2B were grown in Dulbecco's modified Eagle medium (Sigma-Aldrich, St. Louis, MO, USA), supplemented with 10% fetal bovine serum (Sigma-Aldrich), 1% MEM non-essential amino acids (Sigma-Aldrich) and 1% penicillin-streptomycin (Sigma-Aldrich). Cells were grown to ~80% confluency in a humidified chamber at 37 °C, 5% CO_2. Cells were transformed (iAs-T) with sodium arsenite (Sigma-Aldrich) by treating with a low dose of iAs, 0.5 µM, until transformation [1,2]. Time matched control cells were treated with water (non-treated or NT cells) and grown and harvested in parallel with the iAs-transformed cells [1].

Quantification of the Global 5-Methylcytosine Levels in DNA

Genomic DNA was extracted using the DNeasy Blood and Tissue Kit (Qiagen). Total 5-methylcytosine (5-mC) was determined using the 5-mC DNA ELISA kit (Zymo Research Corp., Irvine, CA, USA) per the manufacturer's instruction. The kit utilizes an anti-5-methylcytosine monoclonal antibody that is both sensitive and specific for 5-mC. Briefly, 5-mC was detected using an ELISA-like reaction with the 5-mC antibody.100ng of genomic DNA was used as input for both BEAS-2B lines, as well as the control DNA, which was provided. All samples and methylated standards were measured in triplicate, and reported as an average. The levels of 5-mC in the DNA of the 3 biological samples are reported as the amount of methylated cytosine residues relative to the cytosine genomic content (percent), and data expressed as percent 5-mC in a DNA sample, calculated through a standard curve generated with specially designed controls included in the kit. The graph illustrates triplicate samples ± S.E.M.

qRT-PCR. RNA was isolated from $1x10^7$ BEAS-2B cell with RNeasy MiniKit (Qiagen). 1µg of RNA was used in a reverse transcriptase reaction with iScript Reverse Transcriptase (Bio-Rad)

to prepare cDNA. 25 ng of the cDNA was used in the qRT-PCR reactions. The reaction protocol was as follows: 1) 94 °C 5 min; 2) 94 °C 30 sec; 3) 52-57 °C (dependent on primer pair) 30 sec; 4) 72 °C 45 sec; 5) repeat steps 2-4 for 40 cycles; 6) 72 °C 10 min. Primers for the housekeeping gene (GAPDH) and primers used for genes with DMRs were created for this study using PrimerBank [3-5] and are available in **Supplemental Table 10**. qRT-PCR data was analyzed by the 2^{-ddCt} method, and Student t-tests were performed to determine significance.

Gene Ontology All genes within the top 2000 p-values and a significant DNA methylation change of at least 20% were subjected to a gene ontology annotation using GO-SLIM using the PANTHER [6-9] using terms that had at least 10 genes and p-values <0.05. GSEA software was used to identify other modifications (PRC2 and H3K27me3) that are overrepresented within the gene set [10].

Western Blots. Total nuclear was extracted from 10^7 BEAS-2B cells. Cells were pelleted and washed twice with PBS. The pellet was resuspended in 1mL Hypotonic Solution (20mM Tris 7.4, 10mM NaCl, 3mM $MgCl_2$), and incubated on ice for 15 min. 50µl of 10% NP-40 was added and the mixture vortexed. A nuclear pellet was obtained by centrifugation at 4°C at 3000 rpm for 10 min. Nuclei were resuspended in 100µl 1X RIPA (50 mM Tris-HCl pH8, 150 mM NaCl, 2 mM EDTA pH8, 1% NP-40, 0.5% Sodium Deoxycholate, 0.1% SDS, protease inhibitors) buffer and incubated for 30 min on ice, then centrifuged at full speed, 4°C for 30 min. The concentration of the retained supernatant was quantified with a BCA Kit. Nuclear proteins were resolved on 10% SDS-PAGE gels and run at 130 V until the loading dye reached the end of the gel and then transferred to polyvinylidene fluoride membranes (PVDF) using 65 V for 90 min on ice. Membranes were blocked with 5% Milk + phosphate buffered saline with Tween 20 (PBST) and incubated with primary antibodies (in 0.5% Milk + PBST) overnight at 4 °C. A secondary antibody (α-Rabbit or α-Mouse) was applied the next day and developed by use of ECF (GE-Typhoon FLA9500).

Chromatin Immunoprecipitation. 10^7 cells were resuspended in 1 mL of DMEM. Formaldehyde was added to a final concentration of 0.75%, and cells rotated at 25°C for 10 min. Glycine was added to a final concentration of 125 mM and cells washed three times with cold PBS, then resuspended in ChIP Lysis Buffer (50 mM HEPES-KOH,pH7.5, 140 mM NaCl, 1 mM EDTA, pH 8, 1% Triton X-100, 0.1% Sodium Deoxycholate, 0.1% SDS, protease inhibitors) (750uL per 10^7 cells) and incubated 10 min on ice. Chromatin was disrupted using a biorupter (30 sec on, 30 sec off for a total of 7.5 min – 15 cycles), the chromatin concentration measured and 25 ug used for each immunoprecipitation. The chromatin was loaded with 2 ug of anti-CTCF (Abcam), IgG beads were added, and it was rotated overnight at 4°C. Samples were washed once with low salt (0.1% SDS, 1% Triton X-100, 2 mM EDTA, 20 mM Tris-HCl pH 8, 150 mM NaCl), high salt (0.1% SDS, 1% Triton X-100, 2 mM EDTA, 20 mM Tris-HCl pH 8, 500 mM NaCl), and LiCl wash (0.25 M LiCl, 1% NP-40, 1% Sodium Deoxycholate, 1 mM EDTA, 10 mM Tris-HCl pH 8) buffers. Chromatin was eluted from beads and crosslink was reversed. 2uL of eluate was used for qPCR to determine the change in binding of CTCF to chromatin. Primers used in ChIP analysis are listed in **Supplemental Table 10.**

qPCR. Eluate from the chromatin immunoprecipitation assays was used for qPCR analysis. 2 uL of eluate were used to determine the relative occupancy of CTCF. The protocol used was as follows: 1) 94 °C 5 min; 2) 94 °C 30 sec; 3) 57 °C 30 sec; 4) 72 °C 45 sec; 5) steps 2-4 repeated for 40 total cycles; 6) 72 °C 10 min. Primers used are listed in **Supplemental Table 10.** qPCR data was analyzed by the 2^{-ddCt} method using the IgG beads to normalize the data, and Student t-tests were performed for significance.

1 Riedmann, C. *et al.* Inorganic Arsenic-induced cellular transformation is coupled with genome wide changes in chromatin structure, transcriptome and splicing patterns. *BMC Genomics* **16**, 212, doi:10.1186/s12864-015-1295-9 (2015).
2 Rea, M. *et al.* Quantitative Mass Spectrometry Reveals Changes in Histone H2B Variants as Cells Undergo Inorganic Arsenic-Mediated Cellular Transformation. *Molecular & Cellular Proteomics* **15**, 2411-2422, doi:10.1074/mcp.M116.058412 (2016).
3 Spandidos, A., Wang, X., Wang, H. & Seed, B. PrimerBank: a resource of human and mouse PCR primer pairs for gene expression detection and quantification. *Nucleic Acids Research* **38**, D792-D799, doi:10.1093/nar/gkp1005 (2010).
4 Wang, X. & Seed, B. A PCR primer bank for quantitative gene expression analysis. *Nucleic Acids Research* **31**, e154, doi:10.1093/nar/gng154 (2003).
5 Spandidos, A. *et al.* A comprehensive collection of experimentally validated primers for Polymerase Chain Reaction quantitation of murine transcript abundance. *BMC Genomics* **9**, 633 (2008).
6 Mi, H., Poudel, S., Muruganujan, A., Casagrande, J. T. & Thomas, P. D. PANTHER version 10: expanded protein families and functions, and analysis tools. *Nucleic Acids Research* **44**, D336-D342, doi:10.1093/nar/gkv1194 (2016).
7 Mi, H., Muruganujan, A., Casagrande, J. T. & Thomas, P. D. Large-scale gene function analysis with the PANTHER classification system. *Nat. Protocols* **8**, 1551-1566, doi:10.1038/nprot.2013.092 (2013).
8 Thomas, P. D. *et al.* Applications for protein sequence–function evolution data: mRNA/protein expression analysis and coding SNP scoring tools. *Nucleic Acids Research* **34**, W645-W650, doi:10.1093/nar/gkl229 (2006).
9 Mi, H. & Thomas, P. in *Protein Networks and Pathway Analysis* (eds Yuri Nikolsky & Julie Bryant) 123-140 (Humana Press, 2009).
10 Subramanian, A. *et al.* Gene set enrichment analysis: A knowledge-based approach for interpreting genome-wide expression profiles. *Proceedings of the National Academy of Sciences* **102**, 15545-15550, doi:10.1073/pnas.0506580102 (2005).

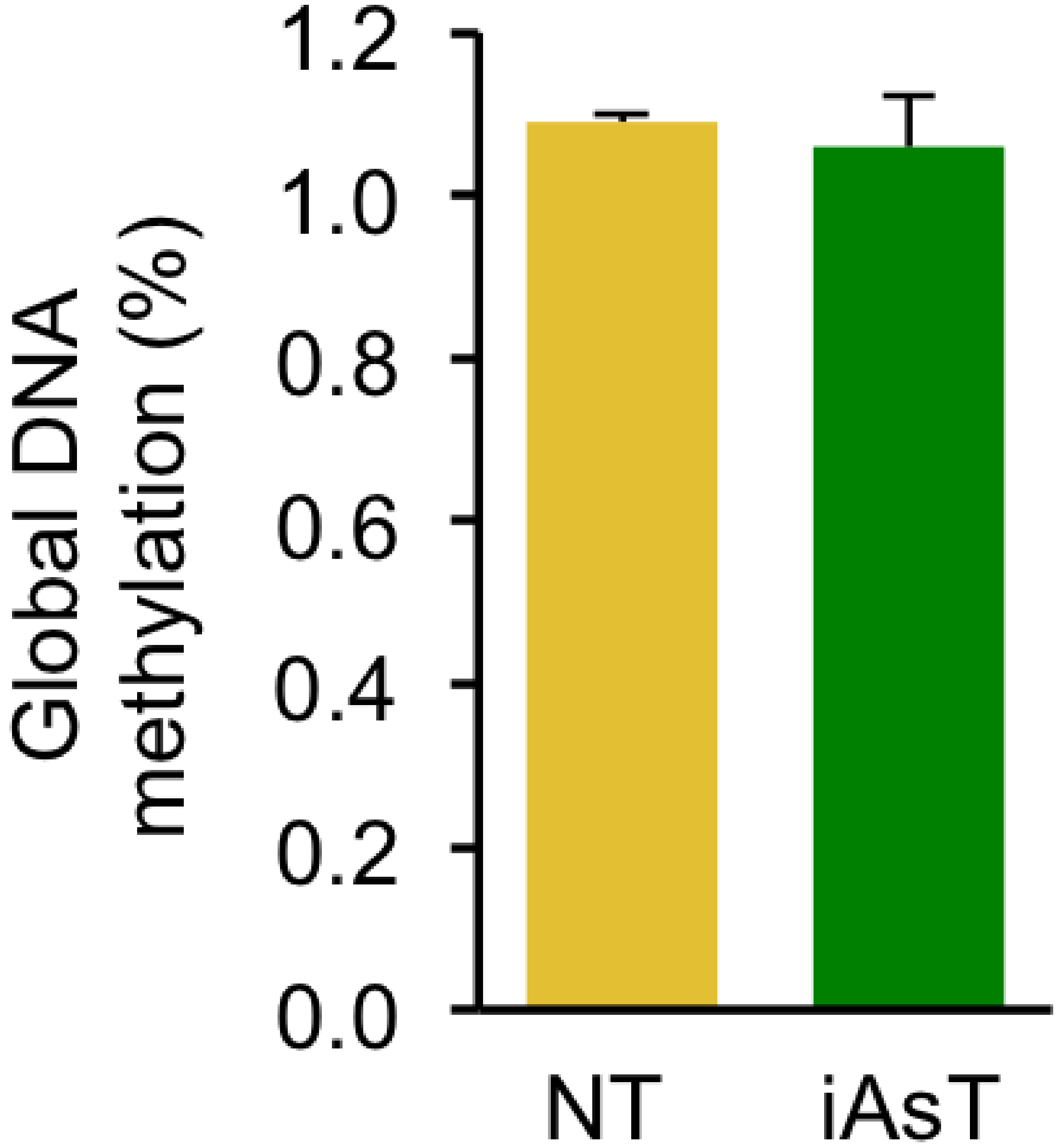

Supplemental Figure 1

Supplemental Figure 1. G**lobal DNA methylation is not significantly reduced with iAs exposure compared to non-treated cells**. An ELISA assay was used to determine the level of DNA methylation in non-treated BEAS 2B cells and in iAs-T BEAS 2B cells. We found a slight, but not significant, decrease in global methylation between iAs-T cells to NT cells. Experiments were performed in triplicate; error bars are S.E.M.

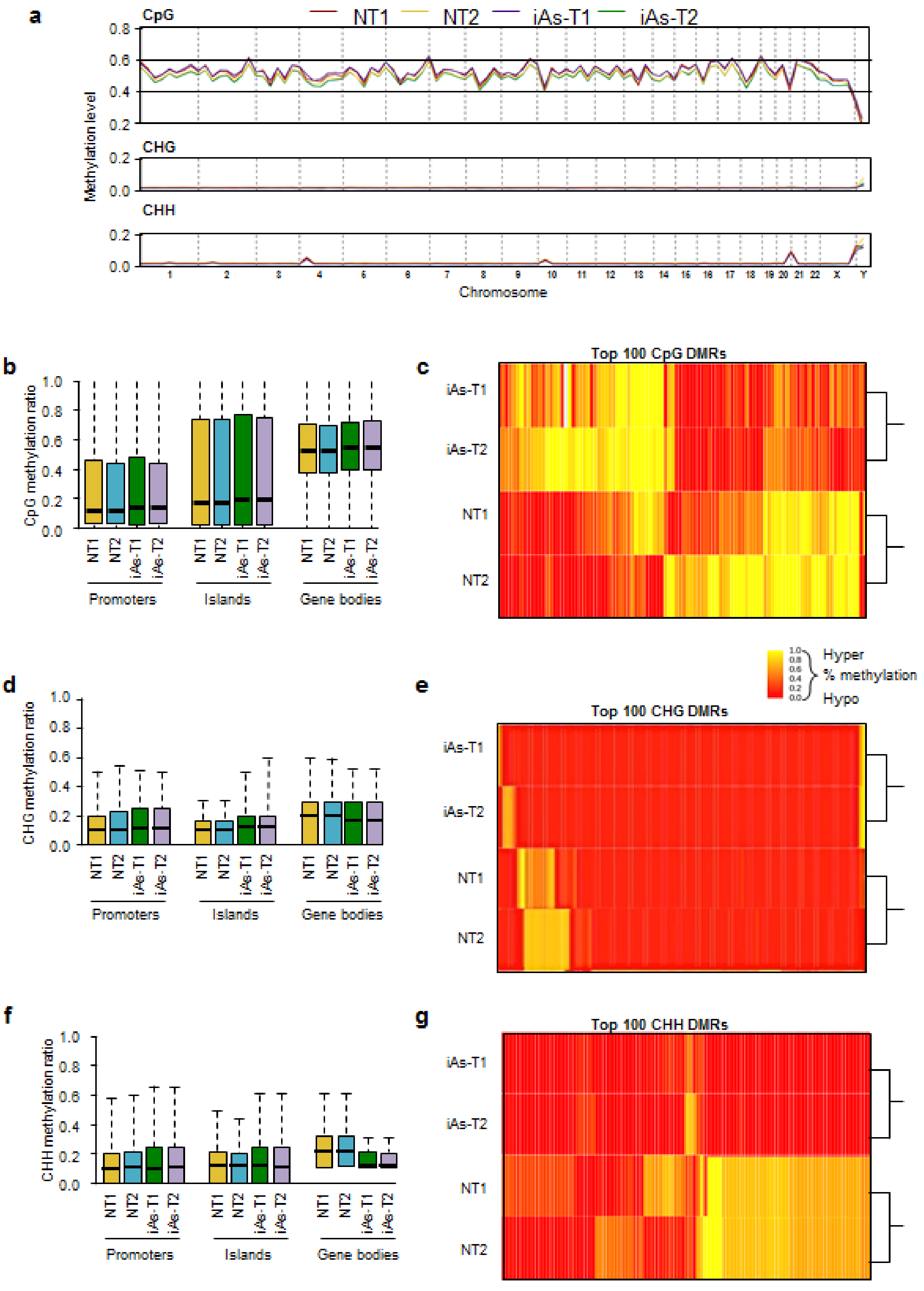

Supplemental Figure 2

Supplemental Figure 2. Correlation analyses of DNA methylation patterns between experimental replicates. (**a**) Global DNA methylation patterns on chromosomes shows there is no difference between the replicates. **b**) There is high concordance amongst the replicates in the CpG methylation pattern changes at different gene regulatory regions. **c**) High concordance was also observed within the topmost 100 changed DNA methylation CpG regions. So too, high reproducibility was observed with the two replicates at CHG (**d**) and CHH (**f**) at gene regulatory sites. This high reproducibility was also observed with the top 100-most DMRs with changes in CHG (**e**) and CHH (**g**) The boxes denote the 25th and 75th percentile (bottom and top of box) and median value (horizontal band inside box) Whiskers indicate the values observed up to 1.5 times the interquartile range above and below the box. Yellow denotes very high methylation (hypermethylation) while red indicates hypomethylation.

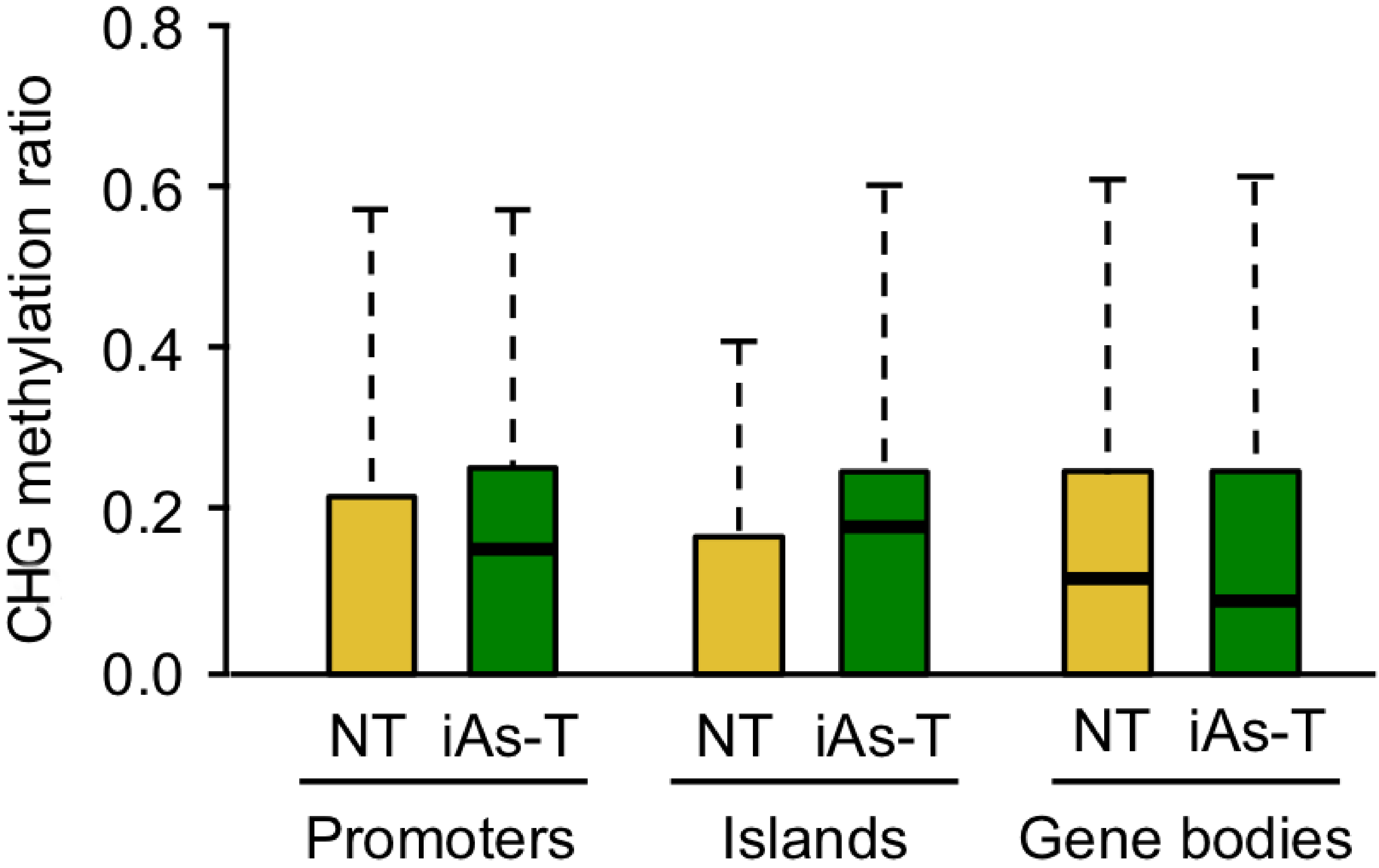

Supplemental Figure 3

Supplemental Figure 3. Analysis of CHG methylation levels at promoters, islands, and gene bodies. Global CHG methylation levels are changed at promoter and/or in gene bodies, but not at CpG Islands (Also see supplemental Figure 2).

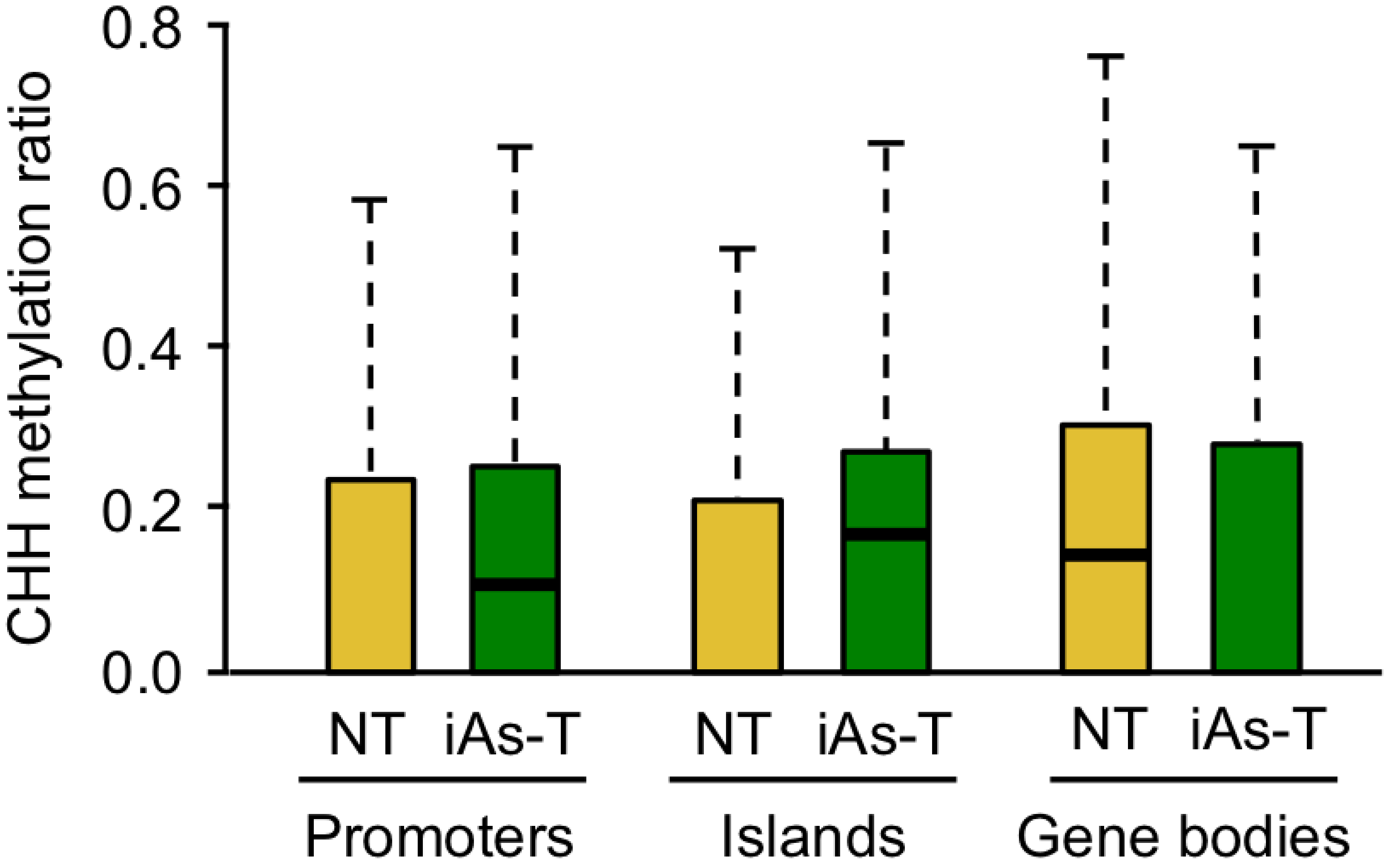

Supplemental Figure 4

Supplemental Figure 4. Analysis of CHH methylation levels at promoters, islands, and gene bodies. Global CHH methylation levels are changed at promoter and/or in gene bodies, but not at CpG Islands. Also see Supplemental Figure 2.

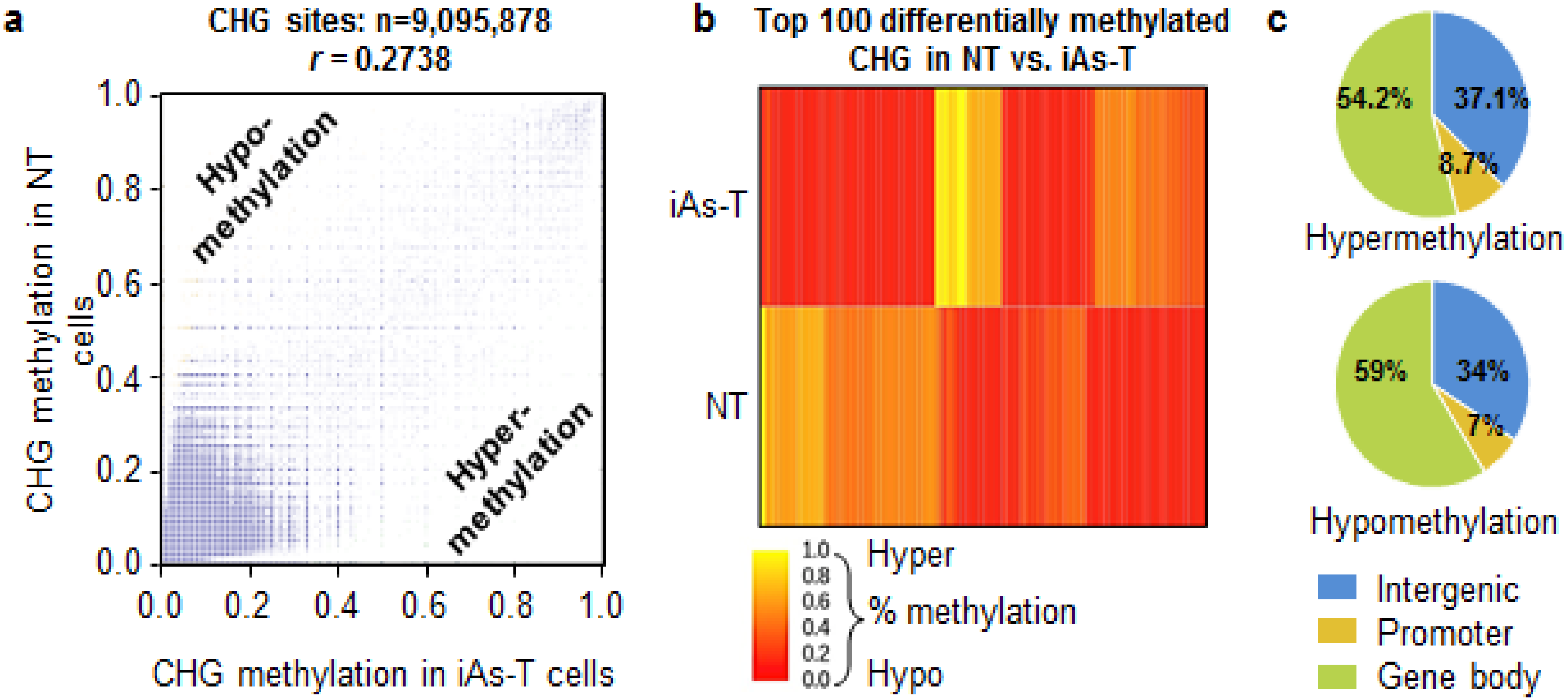

Supplemental Figure 5

Supplemental Figure 5. Differential CHG methylation patterns as cells undergo iAs-induced transformation. (a) Scatterplot of CHG methylation showing low correlation between samples, having a large difference in **(b)** methylation signal of differentially methylated CHG regions. **(c)** Distribution of CpG DMRs across top 2000 sites in hyper- and hypomethylated contexts.

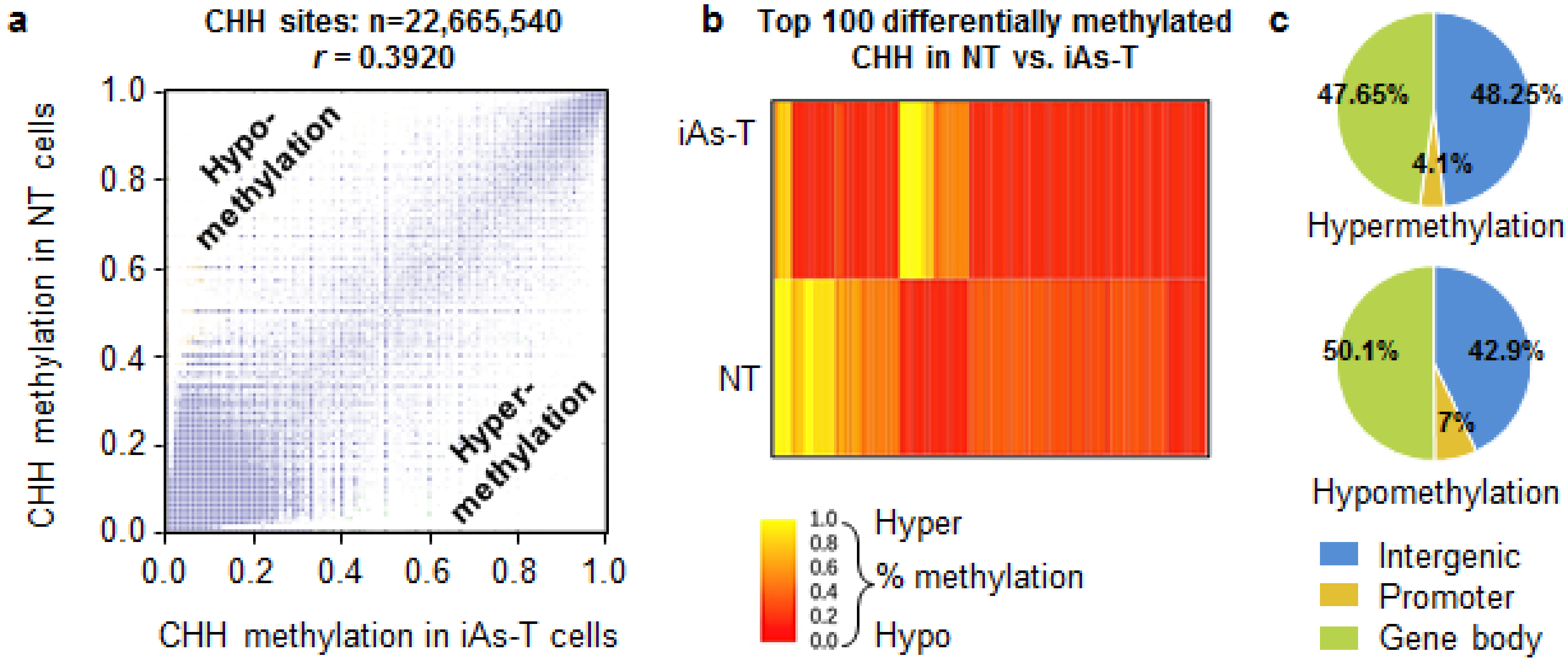

Supplemental Figure 6

Supplemental Figure 6. Differential CHH methylation patterns as cells undergo iAs-induced transformation. (a) Scatterplot of CHH methylation showing low correlation between samples, having a large difference in **(b)** methylation signal of differentially methylated CHH regions. **(c)** Distribution of CGG DMRs across top 2000 sites in hyper- and hypomethylated context

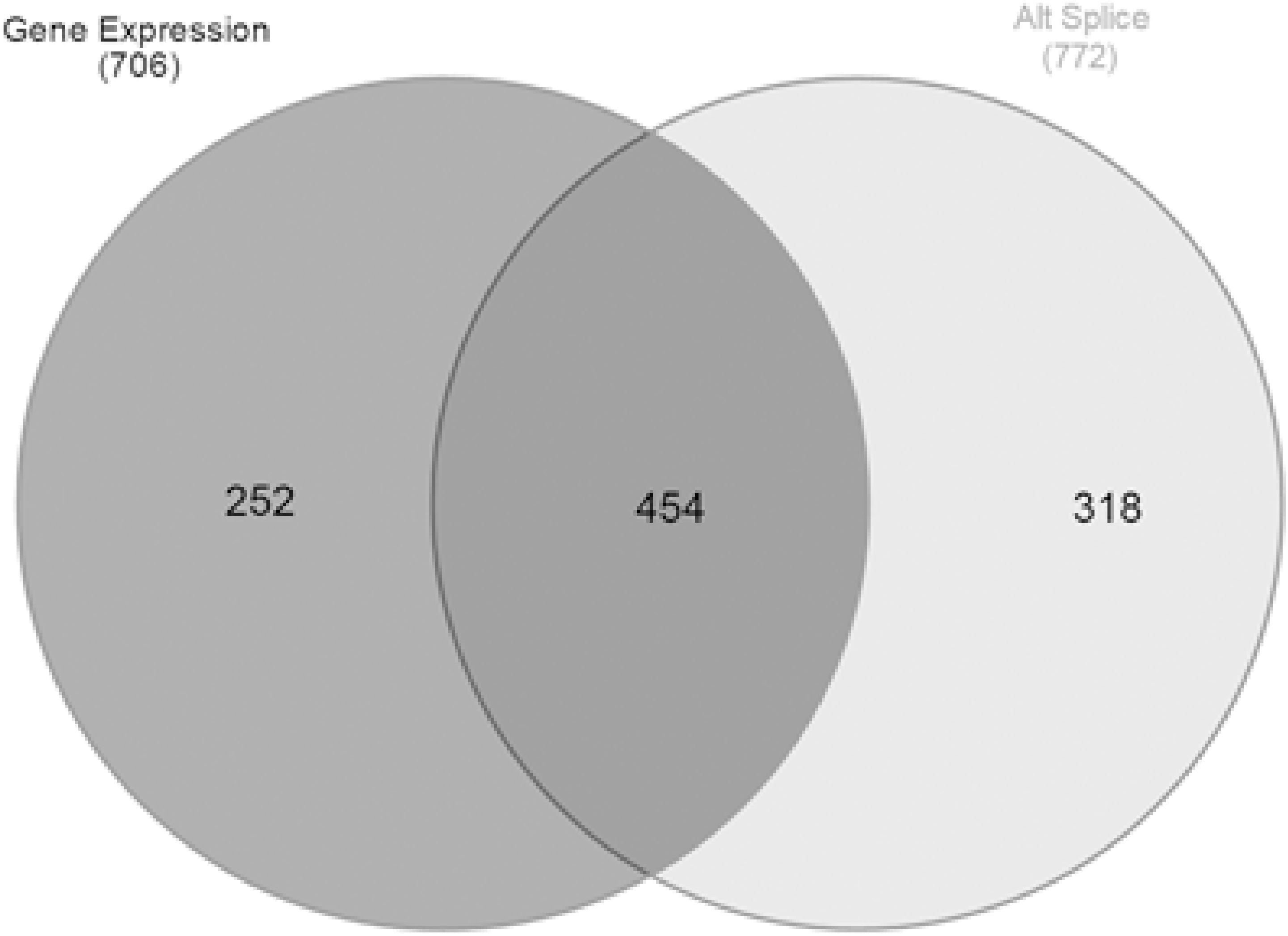

Supplemental Figure 7

Supplemental Figure 7. Overlap of genes with DNA methylation changes that also had changes in gene expression and/or alternative splicing in iAs-T cells. Venn diagram shows the overlap of genes with methylation changes that had changes in Gene Expression (706- left) and/or alternative splicing (772-right). 454 genes had changes in DNA methylation, gene expression and alternative splicing while 252 genes had only changes in gene expression and another 318 had only changes in alternative splicing. GO terms for these genes are in Supplemental Table 9.

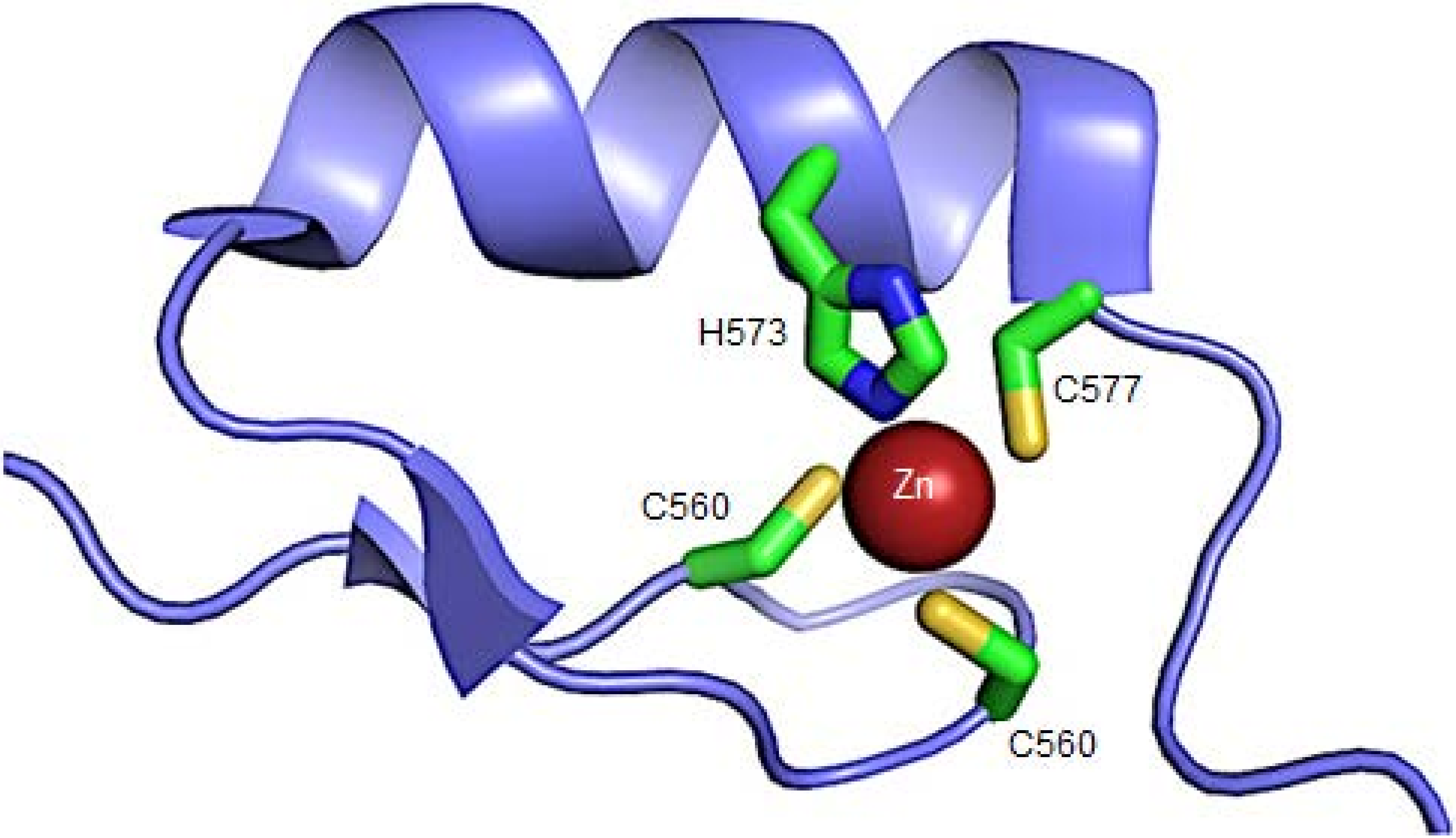

Supplemental Figure 8

Supplemental Figure 8. A stick and ribbon representation of the crystal structure of the 11th zinc-finger of CTCF in complex with a zinc ion (image created from the deposited PDB structure 1X6H). The 1st ten fingers of CTCF have a C2H2 arrangement, while the 11th zinc-finger has a C3H1 arrangement, which would allow iAs to bind instead of Zinc (shown in red), disrupting CTCF's DNA binding.

Supplemental Table 1. Read Coverage of Methyl-seq Replicates of NT and iAs-T Cells.				
**Cell type**	**Total # of reads**	**Unique CpGs**	**Av CpG coverage**	**Bisulfite Conversion rate**
NT_1	38,877,710	8,746,556	7X	99%
iAs-T_1	42,566,995	8,678,428	8X	98%
NT_2	80,369,692	8,400,992	6X	98%
iAs-T_2	85,260,351	9,033,451	8X	98%

Supplemental Table 2. Top 100 CpG Differentially Methylated Genes						
Chromosome	Location	Gene	CGI	meth diff	p-value	classification
chr1	36948570	CSF3R		0.79	1.146E-10	stronglyHypermeth
chr2	99284432	MGAT4A		0.81	8.032E-10	stronglyHypermeth
chr6	25688758	SCGN		0.96	4.802E-09	stronglyHypermeth
chr1	19991675	HTR6	Y	0.88	1.241E-08	stronglyHypermeth
chr2	241562270	GPR35		0.94	5.656E-08	stronglyHypermeth
chr3	180360997	CCDC39		0.89	6.987E-08	stronglyHypermeth
chr1	3321444	PRDM16	Y	0.86	7.27E-08	stronglyHypermeth
chr10	134095552	STK32C	Y	-1	1.035E-07	stronglyHypometh
chr7	126170302	GRM8		0.75	1.575E-07	stronglyHypermeth
chr21	46707145	POFUT2		0.8	2.247E-07	stronglyHypermeth
chr2	163091434	FAP		0.89	5.091E-07	stronglyHypermeth
chr9	9970265	PTPRD		0.77	5.843E-07	stronglyHypermeth
chr10	735362	DIP2C	Y	0.8	5.851E-07	stronglyHypermeth
chr22	19511914	CLDN5	Y	0.8	8.046E-07	stronglyHypermeth
chr18	40516804	RIT2		0.81	0.000001055	stronglyHypermeth
chr18	32073620	DTNA	Y	1	0.000001104	stronglyHypermeth
chr8	143436042	TSNARE1		0.75	0.000001256	stronglyHypermeth
chr21	47573131	FTCD		1	0.00000136	stronglyHypermeth
chr12	133071919	FBRSL1		-1	0.000001684	stronglyHypometh
chr12	68602959	IL26		0.76	0.000002224	stronglyHypermeth
chr10	83634392	NRG3	Y	0.75	0.000002787	stronglyHypermeth
chr2	223169587	CCDC140		0.89	0.000003295	stronglyHypermeth
chr21	45675120	DNMT3L		0.91	0.000003365	stronglyHypermeth
chr8	75232923	JPH1	Y	0.78	0.000003497	stronglyHypermeth
chr4	188916769	ZFP42	Y	0.85	0.000003938	stronglyHypermeth
chr1	7176962	CAMTA1		-1	0.000004079	stronglyHypometh
chr7	50695634	GRB10		0.8	0.000004278	stronglyHypermeth
chr16	78832883	WWOX		0.77	0.000004974	stronglyHypermeth
chr21	47414766	COL6A1		0.94	0.000005761	stronglyHypermeth
chr1	241520041	RGS7		0.78	0.000006692	stronglyHypermeth
chr1	230561389	PGBD5	Y	1	0.00000743	stronglyHypermeth
chr9	39132930	CNTNAP3	Y	0.91	0.000007688	stronglyHypermeth
chr9	977656	DMRT3	Y	0.75	0.000009019	stronglyHypermeth
chr1	1355369	ANKRD65	Y	0.86	0.000009526	stronglyHypermeth
chr4	78671630	CNOT6L		0.88	0.00001243	stronglyHypermeth
chr6	168428398	KIF25		1	0.0000134	stronglyHypermeth
chr22	31091206	OSBP2	Y	-1	0.0000134	stronglyHypometh
chr2	106042582	FHL2		0.92	0.00001369	stronglyHypermeth
chr4	172735018	GALNTL6	Y	0.8	0.00001448	stronglyHypermeth
chr18	77181358	NFATC1		0.75	0.00001795	stronglyHypermeth
chr19	18116795	ARRDC2		1	0.00001843	stronglyHypermeth
chrX	70896591	BCYRN1		0.76	0.00001911	stronglyHypermeth
chr1	237703771	RYR2		0.76	0.00001911	stronglyHypermeth
chr3	71125632	FOXP1		1	0.00001985	stronglyHypermeth
chr15	64686620	TRIP4		1	0.00001985	stronglyHypermeth
chr11	1085929	MUC2		0.8	0.00002027	stronglyHypermeth
chr16	81815600	PLCG2		0.84	0.00002092	stronglyHypermeth
chr4	159629247	ETFDH		0.8	0.00002187	stronglyHypermeth
chr1	40098839	HEYL		1	0.00002285	stronglyHypermeth
chr1	6095480	KCNAB2		-1	0.00002285	stronglyHypometh

chr4	156130070	NPY2R	Y	1	0.00002285	stronglyHypermeth
chr22	51159209	SHANK3	Y	1	0.00002285	stronglyHypermeth
chr1	151104292	SEMA6C	Y	0.77	0.00002294	stronglyHypermeth
chr18	55102007	ONECUT2		-1	0.0000258	stronglyHypometh
chr17	34068908	RASL10B		1	0.0000258	stronglyHypermeth
chr17	45057063	RPRML		1	0.00003142	stronglyHypermeth
chr13	49794900	MLNR	Y	0.77	0.0000315	stronglyHypermeth
chr10	105036768	INA	Y	0.81	0.0000339	stronglyHypermeth
chr10	22634655	SPAG6	Y	0.79	0.0000339	stronglyHypermeth
chr16	70977814	HYDIN		0.75	0.00003656	stronglyHypermeth
chr2	136587728	LCT		-1	0.00003686	stronglyHypometh
chr2	26396899	GAREML	Y	0.75	0.00003803	stronglyHypermeth
chr7	27209615	HOXA	Y	0.86	0.00003844	stronglyHypermeth
chr4	57363635	SRP72		0.8	0.00004161	stronglyHypermeth
chr12	113591593	CCDC42B		0.76	0.00004362	stronglyHypermeth
chr22	29711366	RASL10A	Y	0.75	0.00004716	stronglyHypermeth
chr18	904522	ADCYAP1		0.79	0.00004867	stronglyHypermeth
chr10	135372381	SYCE1		0.85	0.00005309	stronglyHypermeth
chr2	21266714	APOB	Y	1	0.00005387	stronglyHypermeth
chrX	134156526	FAM127C	Y	-1	0.00005387	stronglyHypometh
chr3	14900842	FGD5		-1	0.00005387	stronglyHypometh
chr3	10955503	SLC6A11		1	0.00005387	stronglyHypermeth
chr17	9489678	WDR16		0.76	0.00005937	stronglyHypermeth
chr6	36330512	ETV7		0.8	0.00006119	stronglyHypermeth
chr9	573521	KANK1		0.88	0.00006119	stronglyHypermeth
chr4	17622144	MED28		0.8	0.00006119	stronglyHypermeth
chr4	134072665	PCDH10	Y	0.8	0.00006119	stronglyHypermeth
chr10	118030738	GFRA1	Y	0.75	0.00006617	stronglyHypermeth
chr21	18887956	CXADR		0.83	0.0000673	stronglyHypermeth
chr16	56475395	NUDT21		0.86	0.0000673	stronglyHypermeth
chr11	84825586	DLG2		0.93	0.0000688	stronglyHypermeth
chr19	47960427	SLC8A2	Y	0.86	0.00006934	stronglyHypermeth
chr10	99157935	RRP12		0.75	0.00007041	stronglyHypermeth
chr7	106699782	PRKAR2B		0.83	0.00007097	stronglyHypermeth
chr20	20612924	RALGAPA2		0.8	0.00007139	stronglyHypermeth
chr14	52734656	PTGDR	Y	0.76	0.00007209	stronglyHypermeth
chr1	1893648	KIAA1751		0.84	0.00007432	stronglyHypermeth
chrY	4976514	PCDH11Y		1	0.0000808	stronglyHypermeth
chr12	122688619	B3GNT4	Y	0.78	0.00008368	stronglyHypermeth
chr17	37823761	PNMT	Y	0.82	0.00008457	stronglyHypermeth
chr4	79432289	FRAS1		-1	0.00008741	stronglyHypometh
chr19	48543969	CABP5		0.95	0.00009121	stronglyHypermeth
chr10	72989360	UNC5B		0.82	0.00009281	stronglyHypermeth
chr9	140210349	EXD3		-1	0.0001249	stronglyHypometh
chrX	153095113	PDZD4	Y	-1	0.0001249	stronglyHypometh
chr6	73739381	KCNQ5		-1	0.0001616	stronglyHypometh
chr8	6371105	ANGPT2		-1	0.0001998	stronglyHypometh
chr17	80419253	NARF		-1	0.0001998	stronglyHypometh

chr8	82357708	PMP2		-1	0.0001998	stronglyHypometh
chr5	75807756	IQGAP2		-1	0.0002289	stronglyHypometh

**Supplemental Table 3. Top 100 CHG Differentially Methylated Genes**						
**Chromosome**	**Location**	**Gene**	**CGI**	**meth diff**	**pvalue**	**classification**
chr2	111605411	ACOXL		-0.43	0.000116	stronglyHypometh
chr12	6756495	ACRBP	Y	-0.43	0.000167	stronglyHypometh
chr14	24793760	ADCY4		0.28	0.000431	hypermethylated
chr21	45709561	AIRE		0.27	0.00025	hypermethylated
chr14	23444894	AJUBA		-0.75	0.000722	stronglyHypometh
chr9	135621008	AK8		-0.75	0.000722	stronglyHypometh
chr14	78163890	ALKBH1		0.44	0.000299	stronglyHypermeth
chrX	15872940	AP1S2	Y	-0.67	0.000134	stronglyHypometh
chr19	1467684	APC2	Y	0.61	0.00059	stronglyHypermeth
chr6	101055773	ASCC3		-0.67	0.000547	stronglyHypometh
chr16	28835165	ATXN2L	Y	-0.67	8.46E-05	stronglyHypometh
chr20	17704712	BANF2		-0.5	0.000241	stronglyHypometh
chr12	121680810	CAMKK2		-0.5	0.000709	stronglyHypometh
chr1	7112910	CAMTA1		-0.5	3.71E-07	stronglyHypometh
chrX	41645753	CASK		-0.42	0.000101	stronglyHypometh
chr6	41975685	CCND3		0.29	0.000927	hypermethylated
chr17	37635459	CDK12		-0.52	6.18E-06	stronglyHypometh
chrX	49687584	CLCN5	Y	-0.6	0.000156	stronglyHypometh
chr15	68514018	CLN6		-0.8	0.001032	stronglyHypometh
chr1	24229276	CNR2	Y	0.48	0.000587	stronglyHypermeth
chr7	51292342	COBL		-0.59	3.86E-05	stronglyHypometh
chr14	105944334	CRIP2		0.6	0.000156	stronglyHypermeth
chr10	99790067	CRTAC1	Y	0.27	0.000569	hypermethylated
chr11	45892048	CRY2		-0.5	0.000241	stronglyHypometh
chr2	166383965	CSRNP3		-0.43	5.06E-05	stronglyHypometh
chr19	49871444	DKKL1		-0.4	0.000483	stronglyHypometh
chr16	84191619	DNAAF1		-0.5	0.000948	stronglyHypometh
chr7	21630980	DNAH11		0.47	0.000801	stronglyHypermeth
chr20	62563304	DNAJC5		-0.45	0.000565	stronglyHypometh
chr7	36223005	EEPD1		-0.5	0.000578	stronglyHypometh
chr9	111964067	EPB41L4B		0.8	0.000141	stronglyHypermeth
chr19	17000135	F2RL3		-0.4	0.000483	stronglyHypometh
chr14	86085694	FLRT2		-0.6	2.98E-05	stronglyHypometh
chr16	74605608	GLG1		-0.6	7.61E-05	stronglyHypometh
chr19	2684084	GNG7		-0.52	1.05E-05	stronglyHypometh
chr2	241389236	GPC1		-0.41	1.24E-06	stronglyHypometh
chr7	27243181	HOTTIP		-0.62	0.000851	stronglyHypometh
chr7	27183062	HOXA5	Y	0.27	0.000495	hypermethylated
chr1	87474568	HS2ST1		-0.41	1.7E-05	stronglyHypometh
chr5	63257165	HTR1A	Y	0.26	0.000245	hypermethylated
chr3	9947754	IL17RE		0.28	0.000808	hypermethylated
chr3	197676645	IQCG		0.3	6.91E-05	hypermethylated
chr19	46387855	IRF2BP1	Y	0.36	0.000576	stronglyHypermeth
chr2	227660737	IRS1		-0.4	0.000121	stronglyHypometh
chr3	128870752	ISY1		0.25	0.000416	hypermethylated
chr15	68723445	ITGA11	Y	0.4	0.000431	stronglyHypermeth

chr8	145171164	KIAA1875	Y	0.25	0.000396	hypermethylated
chr10	102988803	LBX1	Y	-0.5	2.9E-05	stronglyHypometh
chr13	39942295	LHFP		-0.43	0.000573	stronglyHypometh
chr1	156892949	LRRC71		-0.46	0.000427	stronglyHypometh
chr14	103886749	MARK3		-0.4	0.000525	stronglyHypometh
chr16	4704027	MGRN1		0.41	0.000721	stronglyHypermeth
chr7	152015994	MLL3		1	0.000333	stronglyHypermeth
chr1	16971308	MST1P2	Y	0.31	0.000578	hypermethylated
chr6	74201599	MTO1		0.46	0.000699	stronglyHypermeth
chr11	20109983	NAV2		0.58	0.000468	stronglyHypermeth
chr10	21455040	NEBL		0.57	1.42E-05	stronglyHypermeth
chr18	77273116	NFATC1	Y	-0.43	0.001088	stronglyHypometh
chr9	127534096	NR6A1		-0.8	0.000285	stronglyHypometh
chr5	37313298	NUP155		-0.4	0.000572	stronglyHypometh
chr6	163265580	PACRG		0.29	0.001064	hypermethylated
chr7	31983814	PDE1C		-0.43	0.000189	stronglyHypometh
chr21	44187744	PDE9A		0.51	0.000546	stronglyHypermeth
chr16	15092392	PDXDC1		-0.42	1.75E-06	stronglyHypometh
chr12	7342406	PEX5	Y	0.29	0.000125	hypermethylated
chr17	65640297	PITPNC1		-0.41	3.86E-10	stronglyHypometh
chr5	57750410	PLK2		0.75	3.09E-05	stronglyHypermeth
chr8	26371164	PNMA2		-0.4	0.000401	stronglyHypometh
chr2	241389216	PP14571		-0.42	0.001005	stronglyHypometh
chr2	45879432	PRKCE	Y	0.5	2.4E-05	stronglyHypermeth
chr6	46458497	RCAN2		-0.45	2.58E-05	stronglyHypometh
chr1	40657038	RLF		-0.44	6.33E-05	stronglyHypometh
chr3	197676650	RPL35A		0.26	0.000442	hypermethylated
chr19	50156160	SCAF1	Y	0.27	0.000821	hypermethylated
chr7	55917972	SEPT14		-0.43	0.000668	stronglyHypometh
chr10	7256394	SFMBT2		-0.41	0.000264	stronglyHypometh
chr12	132259837	SFSWAP		-0.5	0.00102	stronglyHypometh
chr11	62640767	SLC3A2		0.28	0.000104	hypermethylated
chr3	143125557	SLC9A9		-0.67	0.000547	stronglyHypometh
chr22	24962943	SNRPD3		-0.5	0.00102	stronglyHypometh
chr3	181308914	SOX2-OT		-0.44	0.000067	stronglyHypometh
chr2	174818351	SP3		-0.67	0.000417	stronglyHypometh
chr11	120996290	TECTA		0.38	0.000198	stronglyHypermeth
chr14	105070483	TMEM179		0.32	1.28E-06	hypermethylated
chr17	10622849	TMEM220		0.28	0.000658	hypermethylated
chr13	114467298	TMEM255B		-0.67	0.000547	stronglyHypometh
chrX	103217145	TMSB15B	Y	-0.57	0.000346	stronglyHypometh
chr2	218752898	TNS1		0.26	0.000369	hypermethylated
chr1	3657199	TP73		-0.44	0.000907	stronglyHypometh
chr8	140951643	TRAPPC9		0.69	0.001007	stronglyHypermeth
chr12	3014600	TULP3		0.67	0.000255	stronglyHypermeth
chr18	657443	TYMS	Y	-0.42	0.000178	stronglyHypometh
chr9	92254296	UNQ6494		0.3	5.3E-05	hypermethylated
chr3	126234031	UROC1		-0.42	0.00064	stronglyHypometh

chr17	7606350	WRAP53		-0.41	0.000198	stronglyHypometh
chr14	89063139	ZC3H14		-0.55	6.99E-05	stronglyHypometh
chr19	3808523	ZFR2		-0.56	0.000884	stronglyHypometh
chr18	74555974	ZNF236		-0.66	0.000594	stronglyHypometh
chr12	124539446	ZNF664		-0.41	9.35E-05	stronglyHypometh
chr19	13921615	ZSWIM4		0.32	0.000748	hypermethylated

Supplemental Table 4. Top 100 CHH Differentially Methylated Genes						
**Chromosome**	**Location**	**Gene**	**CGI**	**meth diff**	**p-value**	**classification**
chr1	7112911	CAMTA1		-0.58	1.59E-08	stronglyHypometh
chr6	122803886	PKIB		-0.64	1.84E-08	stronglyHypometh
chr5	37313300	NUP155		-0.6	6.47E-06	stronglyHypometh
chr2	51063256	NRXN1		-1	7.43E-06	stronglyHypometh
chr17	6485114	KIAA0753		0.58	1.35E-05	stronglyHypermeth
chr14	55366772	GCH1		-0.57	1.69E-05	stronglyHypometh
chr17	64709368	PRKCA		-0.55	1.97E-05	stronglyHypometh
chr9	6488161	UHRF2		-1	2.58E-05	stronglyHypometh
chr14	86085687	FLRT2		-0.6	2.98E-05	stronglyHypometh
chr17	12599593	MYOCD		-0.6	2.98E-05	stronglyHypometh
chr1	151757626	TDRKH		0.62	3.47E-05	stronglyHypermeth
chr8	27281396	PTK2B		0.75	4.72E-05	stronglyHypermeth
chr16	85668587	GSE1		-1	6.45E-05	stronglyHypometh
chr7	28448788	CREB5	Y	0.78	7.49E-05	stronglyHypermeth
chr13	111563164	ANKRD10		0.42	8.2E-05	stronglyHypermeth
chr16	67633130	CTCF		-0.8	9.18E-05	stronglyHypometh
chr4	25031997	LGI2		-0.67	9.19E-05	stronglyHypometh
chr9	15437567	SNAPC3		0.46	9.36E-05	stronglyHypermeth
chr1	241301970	RGS7		-0.67	0.000111	stronglyHypometh
chr22	28155021	MN1		-0.75	0.000122	stronglyHypometh
chr18	13241360	LDLRAD4		-0.61	0.000127	stronglyHypometh
chr17	61497700	TANC2		-0.54	0.00013	stronglyHypometh
chr19	1924585	SCAMP4	Y	0.67	0.000134	stronglyHypermeth
chr19	3808374	ZFR2		-0.67	0.000142	stronglyHypometh
chr4	83287533	HNRNPD		0.38	0.000144	stronglyHypermeth
chr3	128619339	ACAD9		0.44	0.000155	stronglyHypermeth
chr7	130650836	FLJ43663		-0.56	0.000168	stronglyHypometh
chr4	22477917	GPR125		0.5	0.000176	stronglyHypermeth
chr15	66993814	SMAD6	Y	-0.67	0.000182	stronglyHypometh
chr14	91111449	TTC7B		-0.75	0.000208	stronglyHypometh
chr10	13709169	FRMD4A		-0.56	0.000218	stronglyHypometh
chr12	3014597	TULP3		0.67	0.000255	stronglyHypermeth
chr6	159420946	RSPH3	Y	-0.8	0.000285	stronglyHypometh
chr9	96310501	FAM120A		-0.6	0.000285	stronglyHypometh
chr6	161800920	PARK2		-0.6	0.000285	stronglyHypometh
chr15	76599916	ETFA		0.71	0.000297	stronglyHypermeth
chr16	88973513	CBFA2T3		0.39	0.000319	stronglyHypermeth
chr16	25236922	AQP8		-0.67	0.000323	stronglyHypometh
chr1	156010020	UBQLN4		-0.67	0.000323	stronglyHypometh
chrX	30731098	GK		-1	0.000333	stronglyHypometh
chr7	152015984	MLL3		1	0.000333	stronglyHypermeth
chr17	40046896	ACLY		-0.56	0.000334	stronglyHypometh
chrX	100701834	ARMCX4		0.5	0.000335	stronglyHypermeth
chr19	51504629	KLK8		-0.56	0.000344	stronglyHypometh
chr19	18708433	CRLF1		-0.6	0.000354	stronglyHypometh
chr4	154473185	KIAA0922		-0.6	0.000354	stronglyHypometh

chr7	29924708	WIPF3		-0.6	0.000354	stronglyHypometh
chr16	10635282	EMP2		0.38	0.000355	stronglyHypermeth
chr17	8759497	PIK3R6		0.57	0.000356	stronglyHypermeth
chr6	16719884	ATXN1		-0.67	0.000367	stronglyHypometh
chr2	174818341	SP3		-0.67	0.000417	stronglyHypometh
chr21	45387107	AGPAT3		0.48	0.000447	stronglyHypermeth
chr11	20109974	NAV2		0.58	0.000468	stronglyHypermeth
chr1	63716246	LINC00466		0.5	0.000474	stronglyHypermeth
chr11	858602	TSPAN4	Y	0.5	0.000474	stronglyHypermeth
chr19	50909284	POLD1		-0.57	0.000513	stronglyHypometh
chr17	18215586	TOP3A		0.42	0.000529	stronglyHypermeth
chr16	84191629	DNAAF1		-0.57	0.00053	stronglyHypometh
chr6	101055769	ASCC3		-0.67	0.000547	stronglyHypometh
chr19	18990002	CERS1		0.67	0.000547	stronglyHypermeth
chr16	84651328	COTL1	Y	-0.67	0.000547	stronglyHypometh
chr3	143125581	SLC9A9		-0.67	0.000547	stronglyHypometh
chr3	85919011	CADM2		-0.8	0.000565	stronglyHypometh
chr19	44632264	ZNF225		-0.8	0.000565	stronglyHypometh
chrX	64707999	ZC3H12B		0.4	0.000572	stronglyHypermeth
chr17	32205469	ASIC2		-0.71	0.000624	stronglyHypometh
chrX	15872964	AP1S2	Y	-0.64	0.000642	stronglyHypometh
chr14	23444882	AJUBA		-0.75	0.000722	stronglyHypometh
chr2	25241508	DNAJC27		0.86	0.000722	stronglyHypermeth
chr16	89989108	TUBB3	Y	-0.75	0.000722	stronglyHypometh
chr4	18024200	LCORL	Y	0.67	0.000733	stronglyHypermeth
chr8	18670102	PSD3		-0.67	0.000733	stronglyHypometh
chr14	81942905	SEL1L		-0.67	0.000733	stronglyHypometh
chr3	23903190	UBE2E1		-0.67	0.000733	stronglyHypometh
chr3	130694593	ATP2C1		0.43	0.000782	stronglyHypermeth
chr1	146641998	PRKAB2		0.45	0.000797	stronglyHypermeth
chr22	46853586	CELSR1		0.38	0.00085	stronglyHypermeth
chr7	27243190	HOTTIP		-0.62	0.000851	stronglyHypometh
chr4	2831267	SH3BP2		0.62	0.000851	stronglyHypermeth
chr14	64320298	SYNE2	Y	-0.62	0.000851	stronglyHypometh
chr16	67314597	PLEKHG4		0.67	0.000855	stronglyHypermeth
chr7	2964077	CARD11		0.76	0.000884	stronglyHypermeth
chr2	145225765	ZEB2		0.56	0.00094	stronglyHypermeth
chr19	18468344	PGPEP1		0.67	0.001003	stronglyHypermeth
chr6	158412564	SYNJ2		0.85	0.001032	stronglyHypermeth
chr16	58529331	NDRG4		0.45	0.00106	stronglyHypermeth
chr14	78163878	ALKBH1		0.38	0.001134	stronglyHypermeth
chr1	58531252	DAB1		0.38	0.001174	stronglyHypermeth
chr1	16089953	FBLIM1		0.78	0.001178	stronglyHypermeth
chr9	131773588	SH3GLB2		0.53	0.001235	stronglyHypermeth
chr19	55528204	GP6		0.44	0.001239	stronglyHypermeth
chr22	43534578	MCAT		0.4	0.001318	stronglyHypermeth
chr6	157099843	ARID1B		0.4	0.001409	stronglyHypermeth
chr5	172410993	ATP6V0E1	Y	0.67	0.001412	stronglyHypermeth

chr17	9146166	NTN1		0.67	0.001412	stronglyHypermeth
chr3	142474097	TRPC1		0.4	0.001547	stronglyHypermeth
chr6	160148218	WTAP	Y	0.4	0.001547	stronglyHypermeth
chr1	245380934	KIF26B		0.5	0.001556	stronglyHypermeth
chr3	65773011	MAGI1		0.6	0.00156	stronglyHypermeth
chr15	71056082	UACA	Y	0.42	0.001578	stronglyHypermeth

Supplemental Table 5. Top 2000 non-CpG Differentially Hypermethylated Genes		
**GO Term**	**Number of Genes**	**p-value**
Cell communication	241	4.68E-07
Intracellular Signal transduction	213	4.45E-05
ATP Binding	130	6.44E-05
Neurogenesis	125	2.31E-03
H3K4me3 and H3K27me3	117	9.30E-45
Cell adhesion	110	7.58E-03
Kinase	90	4.72E-05
SUZ12 targets	87	4.79E-25
H3K27me3	82	5.04E-20
Cell Migration	78	1.19E-03
Cytoskeletal Protein Binding	77	1.69E-02
Nervous System Development	77	3.95E-03

Supplemental Table 6. Top 2000 non-CpG Differentially Hypomethylated Genes		
**GO term**	**Number of Genes**	**p-value**
Regulation of Gene expression	203	2.17E-06
Metal Ion Binding	192	2.33E-07
Neurogenesis	118	1.78E-10
Cell Proliferation	94	1.95E-03
Cell Adhesion	76	4.41E-04
Neuron Differentiation	74	2.07E-06
H3K4me3 and H3K27me3	71	9.79E-21
Protein Kinase Binding	57	2.78E-07
Cell-Cell adhesion	52	3.79E-02
Chromatin Binding	49	1.34E-05
Neuron Projection Development	48	9.10E-04
H3K27me3	37	1.30E-14
Axon Guidance	23	4.44E-03

Supplemental Table 7. Promoter Differentially Regulated Genes with iAs exposure.

Gene Symbol	Location	Methyl Diff	CGI	P-value	Expression Change	
GDF6	Chr8:97173227	1	Y	5.387E-05	-2.63	Hyper
GIPC2	Chr1:78511849	0.8	Y	2.88e-04	-1.75	Hyper
GPRIN1	Chr5:176036479	1	Y	2.58e-04	-1.82	Hyper
HEYL	Chr1:40105474	0.77	Y	1.575E-07	-2.77	Hyper
NRG3	Chr10:83634392	0.88	Y	3.753e-03	-1.88	Hyper
DIABLO	Chr12:122711313	-0.79	Y	6.22e-03	1.51	Hypo
PTPRJ	Chr11:48001742	-0.74	Y	5.72E-08	1.46	Hypo
KCNK17	Chr6:39282185	-0.82	Y	2.69E-05	1.04	Hypo
OSPB2	Chr22:31091206	-1	Y	1.25e-04	12.1	Hypo
OLFM1	Chr9:137980169	-0.86	Y	1.14E-04	2.52	Hypo

Supplemental Table 8. Gene Body Differentially Regulated Genes with iAs exposure.

Gene Symbol	Location	Methyl Diff	CGI	P-value	Expression Change	
GALNT13	Ch2: 155062765	0.75	N	8.819e-09	+1.34	Hyper
SEMA5B	Chr3:122641030	0.86	Y	5.789e-08	+0.49	Hyper
CLEC11A	Ch19:51227723	0.78	Y	1.29e-06	+1.5	Hyper
NCAM2	Chr21:22587939	0.71	N	2.00e-04	+8.2	Hyper
PCDH10	Chr4:134072665	0.8	Y	6.119E-05	+4.2	Hyper
CA10	Chr17:50217743	-0.79	N	5.33E-07	-1.38	Hypo
DSCAM	Chr21:41883745	-0.75	N	5.97E-06	-3.22	Hypo
IGSF9B	Chr11:133788135	-0.73	Y	5.35E-07	1.18	Hypo
GPR160	Chr3:169773611	-0.81	N	1.06E-07	-1.23	Hypo
PCDH19	chrX:99663955	-0.86	Y	1.41E-04	-5.26	Hypo

Supplemental Table 9. Gene Ontology of overlapping Genes		
Overlap Genes of DMRs and Gene Expression Changes (706)		
GO Term	Number of Genes	p-value
Actin Binding	38	6.81E-05
Cadherin Binding	31	9.46E-05
ATP binding	97	1.15E-06
Neurogenesis	99	5.83E-07
Axon Guidance	23	9.97E-03
Regulation of Cell motility	53	3.21E-03
Cell-cell adhesion	60	2.23E-03
Cell Adhesion	92	8.70E-08
Overlap Genes of DMRs and Alternative Splicing (318)		
Nucleotide binding	64	2.64E-02
Nervous System Development	65	1.86E-03
Signal Transduction	51	6.86E-03
Cell Differentiation	89	2.97E-04
Cell Projection	48	8.78E-16
Neurogenesis	41	8.63E-15
Cell Localization	27	4.62E-11

Supplemental Table 10. Primers Used in this Study

Primer Name	Primer Sequence (5’-3’)	Reference	PrimerBank ID	Annealing Temperature (°C)
GAPDH 197F	GGAGCGAGATCCCTCCAAAAT	PrimerBank	378404907c1	50-60
GAPDH 197R	GGCTGTTGTCATACTTCTCATGG			
GALNT13 141F	TCGTTCCCCACACTATCTACTC	PrimerBank	145309312c2	57
GALNT13 141R	CCAGAGCGTTCTTCCATCCTA			
SEMA5B 228F	CCGTGGGTCTCTAACTTCACC	PrimerBank	373432661c1	57
SEMA5B 228R	GACTCGCACGTAGTTCTGACA			
CLEC11A 142F	CTGCCGGAACTGTTGAGGG	PrimerBank	2257695a1	55
CLEC11A 142R	CCCAGGATGTAAGTGACGATGT			
GIPC2 176F	GGCGCGTTTGAAATCTCGC	PrimerBank	41393578c1	52
GIPC2 176R	GTGAGACCAAGTGAATCCTCAG			
PTPRJ 149F	ACAGAGCCGATCCCAGTTTCT	PrimerBank	148728159c3	55
PTPRJ 149R	CTTGAGTCTTGAGTCAACTCCTC			
GPR160 82F	CCAGCCATCTACCAAAGCCTG	PrimerBank	197304798c1	52
GPR160 82R	GCCAGTAACTCTGAATGCTGACA			
CA10 119F	TCATCGTCTGCATATCAGCTCA	PrimerBank	130979953c1	52

CA10 119R	GTTCACCAATCCCCAGAAAGAAG			
IGSF9B 105F	AGAGGAGCCCGAGTTTGTGA	PrimerBank	148886751c1	57
IGSF9B 105R	CACTCTACGACATAGGGTGGG			
NCAM2 118F	GGGGTTGCTTGTCAGTAGC	PrimerBank	316659209c1	52
NCAM2 118R	TTCAGGTTCACCAATCGCTGT			
PCDH10 87F	TGGATGGTGGAAGGAGTCTTT	PrimerBank	14589915c1	55
PCDH10 87R	TTCAGCGATATTCCCCACGAA			
DSCAM 124F	ATCAGACCCAGCGAACTCAG	PrimerBank	45827724c3	57
DSCAM 124R	CCAGCGGTAATCTGGCTCAG			
PCDH19 141F	CTTTGGCGTGCAGACTTACGA	PrimerBank	157426844c2	50
PCDH19 141R	CGGAAGCTGTAGTGCGACT			
HEYL 77F	GGAAGAAACGCAGAGGGATCA	PrimerBank	105990530c1	57
HEYL 77R	CAAGCGTCGCAATTCAGAAAG			
GPRIN1 225F	AAAGCAGGCCGATTCCACTTC	PrimerBank	112821680c1	57
GPRIN1 225R	TCCTTCCTCGGTGACACTGTA			
GDF6 96F	CACGAGTACATGCTGTCAATCT	PrimerBank	188219580c1	57

GDF6 96R	CGTATTAGCCGACTTGGAAGAC			
NRG3 110F	ACGACGACATATTCCACAGAGC	PrimerBank	260166671c1	57
NRG3 110R	CCGGTCAGGGTTTCGATCAC			
KCNK17 139F	GATCCGGGATGTCGTCCAAG	PrimerBank	205361106c1	57
KCNK17 139R	GCTCAGGTTGCCATAGCCAAT			
OSBP2 193F	TCGTCGCTGTTCACGGTTG	PrimerBank	75905817c1	52
OSBP2 193R	CGGACACAGGTTCCGATCTTG			
OLFM1 53F	AGGGCAGGTGTATCTGCAC	PrimerBank	138175804c1	50
OLFM1 53R	CCGTGAACACATGGTCTGCT			
DIABLO 127F	CGCGCAGCGTAACTTCATTC	PrimerBank	218505810c1	55
DIABLO 127R	CCAAAGCCAATCGTCACAGTTTT			
PTPRJ BSF	GTTYGGGGGTTGGGGGATAGAATATAATG	Zymo Bisulfite Primer Seeker		58
PRPRJ BSR	CCCAACCCTCCCACATTCCTAAAACTTAAAACCC			
NRG3 BSF	TGATTTTYGATTTTTTTGTTTGTTTGTTGGG	Zymo Bisulfite Primer Seeker		50
NRG3 BSR	CATTCCRACACAAAAAACCTACCTAAC			
GIPC2 BSF	GYGGTTGTTATTGGAGGTTGTTTTTATTTG	Zymo Bisulfite Primer		60

		Seeker		
GIPC2 BSR	TCTAAACRTAAAACTCCTAAATACTAAAA AAACCCTCC			
DNMT1 85F	AGGCGGCTCAAAGATTTGGAA	PrimerBank	195927036c1	52
DNMT1 85R	GCAGAAATTCGTGCAAGAGATTC			
DNMT3A 151F	CCGATGCTGGGGACAAGAAT	PrimerBank	12751473a1	52
DNMT3A 151R	CCCGTCATCCACCAAGACAC			
DNMT3B 154F	AGGGAAGACTCGATCCTCGTC	PrimerBank	333440486c1	52
DNMT3B 154R	GTGTGTAGCTTAGCAGACTGG			
CTCF 81F	CAGTGGAGAATTGGTTCGGCA	PrimerBank	300388141c1	57
CTCF 81R	CTGGCGTAATCGCACATGGA			
DNMT1ctcfF1	TCCACCCATCTGCAGGTCTG			57
DNMT1ctcfR1	GTGTTCCCCAGGAAACCAGCC			
DNMT3ActcfF1	TAGAGCCCTGAGCGCTCCATG			57
DNMT3ActcfR1	ACTGGAAGACTGAAAGATTTC			
DNMT3BctcfF2	GCCGCAGCCCGCGTGGACG			57
DNMT3BctcfR2	GCGAATCTCCGGCGCCGATCGC			

OSBP2 BSF	GGGTYGGAGTTTAAGTTTTAGTTTTAG	Zymo Bisulfite Primer Seeker		50
OSBP2 BSR	CTATCCTAATCRCAAAAAAAACAAAAAC TTTAAC			
IGSF9BASF	TTGGACAAGCAGGTGAGTC			57
IGSF9BASR1	ATGACCTGCACAGCTTTTG			
IGSF9BASR2	GGCCAGTCTTCATGATAAGG			
SEMA5BexonAF	TGCTATCTCCCAGGCTTTCAA			54
SEMA5BexonAR	CAGGTTCTCGTTGGGACCG			
